# PPARα and PPARγ activation is associated with pleural mesothelioma invasion but therapeutic inhibition is ineffective

**DOI:** 10.1016/j.isci.2021.103571

**Published:** 2021-12-04

**Authors:** M. Lizeth Orozco Morales, Catherine A. Rinaldi, Emma de Jong, Sally M. Lansley, Joel P.A. Gummer, Bence Olasz, Shabarinath Nambiar, Danika E. Hope, Thomas H. Casey, Y. C. Gary Lee, Connull Leslie, Gareth Nealon, David M. Shackleford, Andrew K. Powell, Marina Grimaldi, Patrick Balaguer, Rachael M. Zemek, Anthony Bosco, Matthew J. Piggott, Alice Vrielink, Richard A. Lake, W. Joost Lesterhuis

**Affiliations:** 1 School of Biomedical Sciences, University of Western Australia, Crawley, WA 6009, Australia; 2 National Centre for Asbestos Related Diseases, Nedlands, WA 6009, Australia; 3 Centre for Microscopy Characterisation and Analysis, Nedlands, WA 6009, Australia; 4 Telethon Kids Institute, University of Western Australia, West Perth, WA 6872, Australia; 5 Institute for Respiratory Health, Nedlands, WA 6009, Australia; 6 School of Science, Department of Science, Edith Cowan University, Joondalup, WA 6027, Australia; 7 UWA Medical School, The University of Western Australia, Crawley, WA 6009, Australia; 8 School of Molecular Sciences, University of Western Australia, Crawley, WA 6009, Australia; 9 School of Veterinary and Life Science, Murdoch University, Murdoch, WA 6150, Australia; 10 Department of Anatomical Pathology, PathWest Laboratory Medicine, QEII Medical Centre, Nedlands, WA 6009, Australia; 11 Centre for Drug Candidate Optimisation, Monash Institute of Pharmaceutical Sciences, Monash University, Parkville, VIC 3052, Australia; 12 IRCM, Institut de Recherche en Cancérologie de Montpellier, Montpellier 34090, France

**Keywords:** Biological sciences, Metabolomics, Transcriptomics

## Abstract

Mesothelioma is a cancer that typically originates in the pleura of the lungs. It rapidly invades the surrounding tissues, causing pain and shortness of breath. We compared cell lines injected either subcutaneously or intrapleurally and found that only the latter resulted in invasive and rapid growth. Pleural tumors displayed a transcriptional signature consistent with increased activity of nuclear receptors PPARα and PPARγ and with an increased abundance of endogenous PPAR-activating ligands. We found that chemical probe GW6471 is a potent, dual PPARα/γ antagonist with anti-invasive and anti-proliferative activity *in vitro*. However, administration of GW6471 at doses that provided sustained plasma exposure levels sufficient for inhibition of PPARα/γ transcriptional activity did not result in significant anti-mesothelioma activity in mice. Lastly, we demonstrate that the *in vitro* anti-tumor effect of GW6471 is off-target. We conclude that dual PPARα/γ antagonism alone is not a viable treatment modality for mesothelioma.

## Highlights

• Pleural location stimulates mesothelioma invasion and proliferation
• PPARα and PPARγ activation is associated with pleural mesothelioma invasion
• Chemical probe GW6471 is a potent PPARα/γ antagonist *in vitro*
• Dual inhibition of PPARα/γ does not have anti-mesothelioma activity *in vivo*

## Graphical abstract

**Figure:**
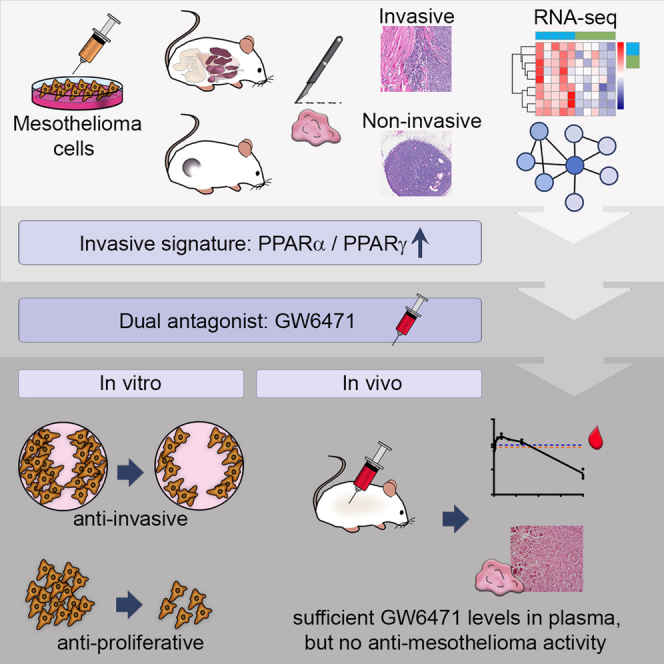

## Introduction

Mesothelioma is a malignancy that develops in the pleura and occasionally at other serosal surfaces, such as the peritoneum, pericardium, and tunica vaginalis (Yap et al., 2017). Mesothelioma is essentially caused by asbestos exposure, and even though asbestos has been banned in most developed countries, it remains present in the urban environment, and many developing countries still use asbestos in manufacturing and building (Gupta and Joshi, 2004). With an average latency period of 35–40 years between asbestos exposure and diagnosis, mesothelioma remains a serious problem due to its high mortality rate, with a five-year survival rate of less than 10% (Mott, 2012). Metastasis does occur, but unlike many other cancers, it does not dominate the clinical course of the disease (Finn et al., 2012). Instead, local invasion into surrounding tissues, such as the chest wall, lungs, heart, and diaphragm causes most morbidity (Robinson and Lake, 2005).

Cancer invasion is associated with the activation of different signaling pathways in tumor cells and the tumor microenvironment, allowing the cancer cells to migrate into neighboring tissues (Friedl and Alexander, 2011). In mesothelioma, several cancer cell-intrinsic factors have been linked to invasion, such as epithelial-to-mesenchymal transition (Mutsaers, 2004), or overexpression of ADAM10 (Sépult et al., 2019) and calretinin (Wörthmüller et al., 2018). Invasion is also associated with upregulated signaling pathways such as hepatocyte growth factor (Gaudino et al., 2014), Ror-family proteins (Saji et al., 2018), extracellular signal-regulated kinases (Tamminen et al., 2015), and focal adhesion kinase (Padval et al., 2014). Extrinsic factors in the tumor microenvironment, including the surrounding stroma, promote tumor invasion, by providing a scaffold for cancer-associated fibroblasts and evasion of anti-tumor immunity (Chu et al., 2019). Moreover, mesothelioma cells produce fibronectin, laminin, and type IV collagen, which stimulate chemotaxis and haptotaxis, promoting tumor invasion (Aota et al., 1991; Klominek et al., 1993).

Tumor progression, metastasis, migration, and invasion occur in the context of altered cancer cell metabolism, including upregulation of glutamine metabolism (Wang et al., 2010), adipocyte-mediated transfer of lipids to cancer cells (Nieman et al., 2011), increased extracellular acidification, and downstream proteolytic activity of matrix metalloproteinases (Rothberg et al., 2013). Additionally, epithelial-to-mesenchymal transition activation has been associated with altered mitochondrial metabolism, glycolysis, and lipid metabolism in other cancers (Liu et al., 2017; Sánchez-Martínez et al., 2015; Nath et al., 2015). However, for mesothelioma, the role of metabolic changes in the development and progression of the disease have not been well characterized, and to date no treatments have been developed that effectively target mesothelioma invasion.

Here, we aimed to identify regulators of mesothelioma invasion by comparing transcriptomic and metabolomic data from invasive and non-invasive murine mesothelioma tumors, in combination with *in vitro* invasion and proliferation assays using chemical probes and knockout cell lines, to drive the development of novel approaches for the treatment of this fatal cancer.

## Results

### Mesothelioma cells are more invasive and divide more rapidly in the pleural cavity compared to the subcutaneous space

**Figure 1:**
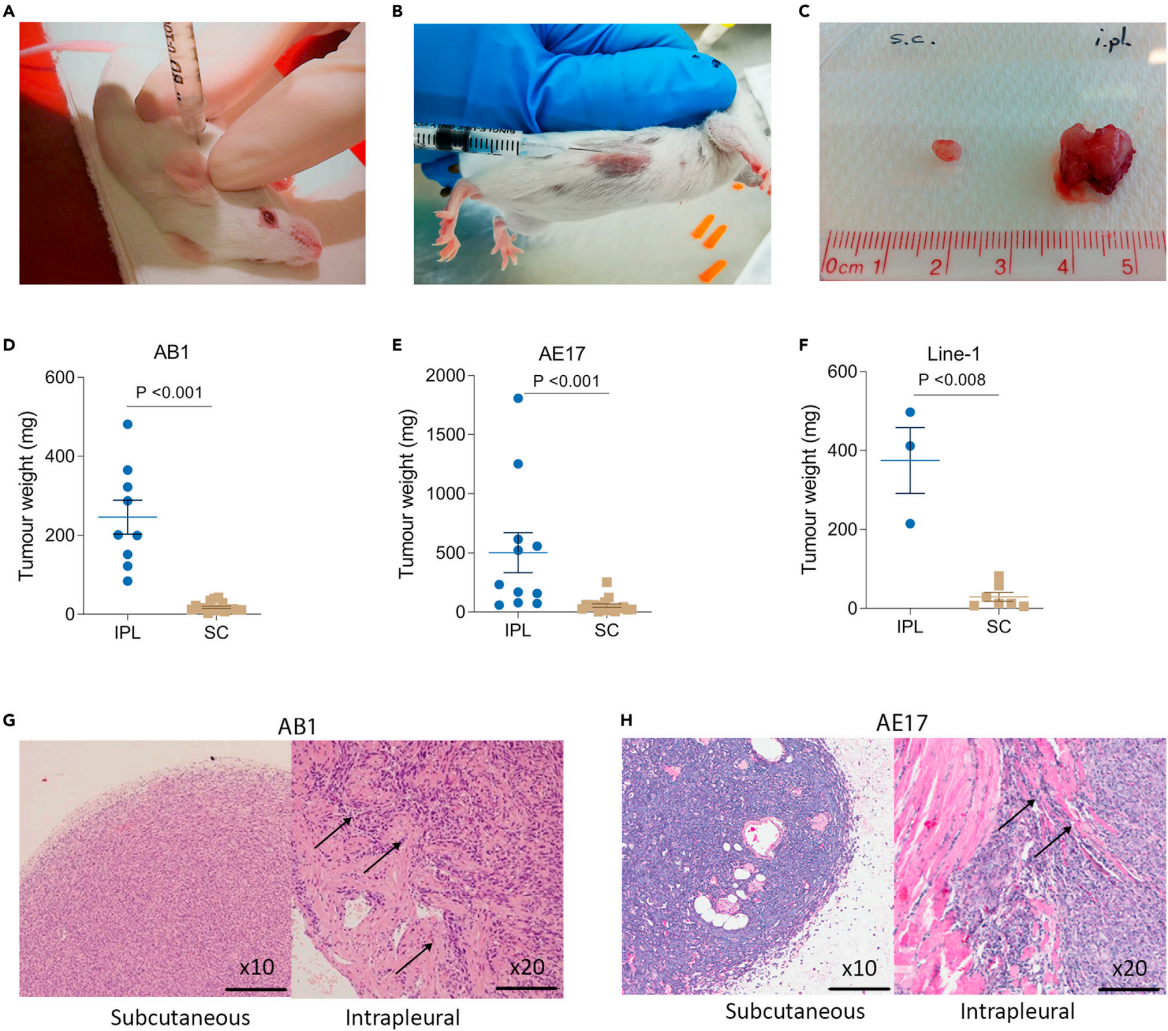
Mesothelioma cells are more invasive and divide more rapidly in the pleural cavity compared to the subcutaneous space Tumor inoculation in the (A) intrapleural site and (B) subcutaneous site in BALB/c mice. (C) Subcutaneous (left) and intrapleural (right) tumors were harvested at day 10. (D–F) Weights from IPL and SC tumors with (D) AB1 cell line in BALB/c mice (E) AE17 cell line in C57BL/6 mice, and (F) Line-1 cell line in BALB/c mice. Data are means ± SEM from three independent sets of experiments. Student's t-test (D) p <0.001 (E) p <0.001, and (F) p <0.008. (G and H) IPL and SC tumors from (G) AB1 and (H) AE17 cell lines were stained with hematoxylin and eosin. Arrows depict tumors penetrating surrounding tissues. Scale bar = 100 μm.

Inoculation of mesothelioma cells into the pleural space of syngeneic mice provides a more clinically relevant model when compared with the often used subcutaneous site. To compare the growth rate of the same cancer cell line in these different environments, we inoculated BALB/c and C57BL/6 mice with the cell lines AB1 and AE17, respectively, in the two different body compartments, using a fixed number of cells from the same culture (Figures 1A and 1B). Tumors were harvested 10 days after inoculation and weighed (Figures 1C,S1A, and S1B). The mass of intrapleural (IPL) tumors was significantly higher than that of subcutaneous (SC) tumors (Figures 1D and 1E; p <0.001). To test whether the increase in tumor size in the pleural cavity was particular to cancers of mesothelial origin, we repeated the experiment with the lung cancer cell line Line-1 and again found bigger tumors in the pleural cavity (Figure 1F; p <0.008), suggesting that the increased tumor growth is a feature of the environment rather than the cancer cell type.

Morphologically, SC tumors were characterized by a pseudo-capsule and they did not invade into other tissues, while the IPL tumors had no capsule and showed clear signs of invading the adjacent tissues, including the diaphragm, intercostal muscles (away from the injection site), the lungs and in some cases, even showed transmural penetration of the heart (Figures 1G and 1H). Together, these results indicate that the pleural microenvironment induces a highly invasive phenotype in mesothelioma cells that is not present in the subcutaneous space.

### PPARα and γ signaling are associated with invasive pleural mesothelioma development

**Figure 2:**
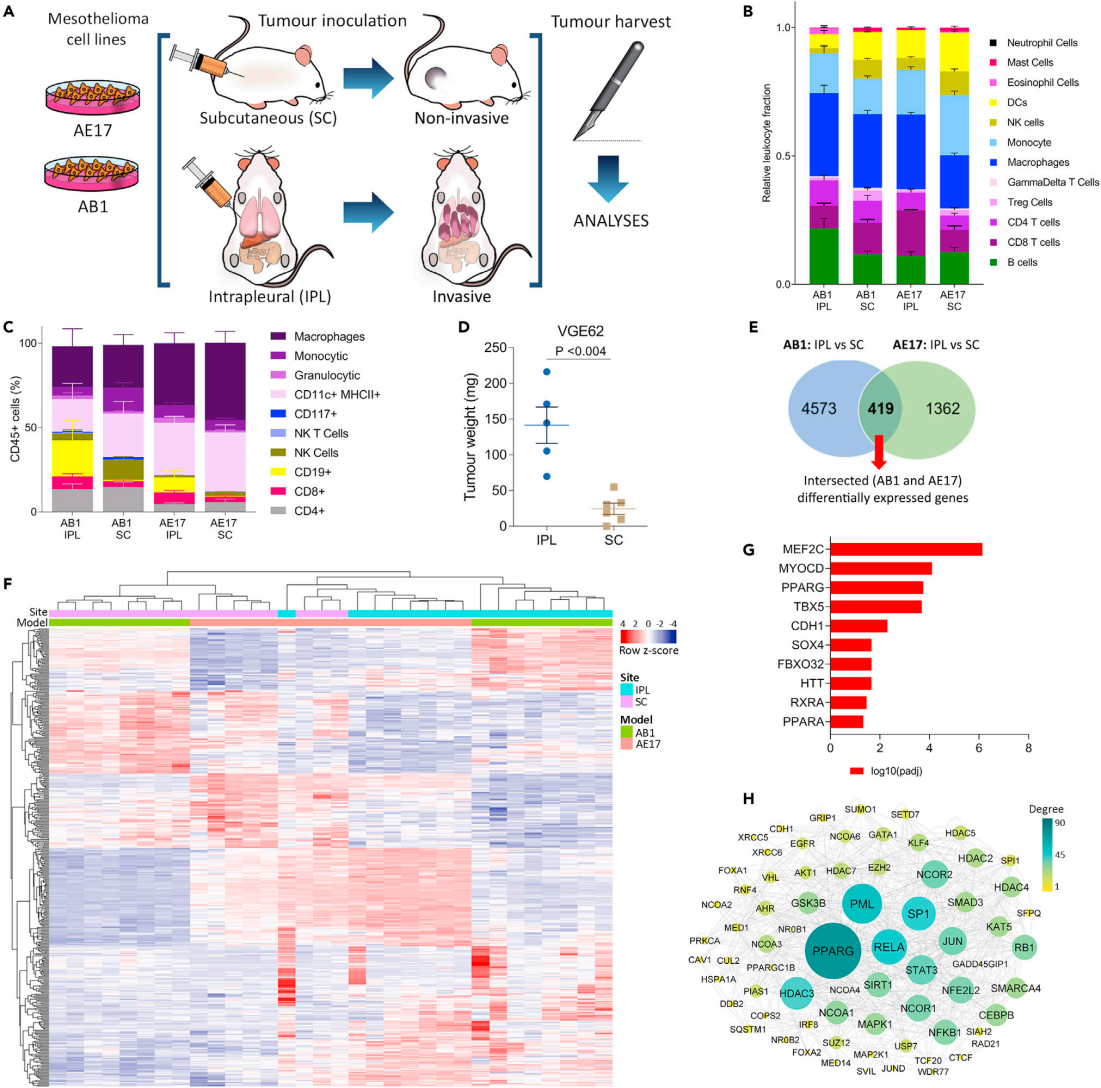
PPARα and γ signaling are associated with invasive pleural mesothelioma development (A) Experimental design, n (mice) = 8 per group. (B) CIBERSORT analysis from AB1 SC, AB1 IPL, AE17 SC, and AE17 IPL groups, n = 8 per group. (C) Flow cytometry of dissociated tumors from AB1 SC (n = 8), AB1 IPL (n = 8), AE17 SC (n = 5), and AE17 IPL (n = 5) bearing mice. (D) Weight (mg) from IPL and SC tumors with VGE62 cell line in NSG mice. Data are presented as means ± SEM. Student's t-test, p <0.004. (E) Venn diagram with the number of differentially expressed genes between IPL and SC tumors from AB1 (4573) and AE17 (1362) models. (F) Unsupervised-hierarchical clustering of intersected differentially expressed genes. (G) Upstream regulator analysis of intersected differentially expressed genes. (H) Graphical reconstruction of network based on the turquoise module obtained from the WGCNA analysis (Figure S2). Node degree is shown as size and color.

To further characterize the underlying mechanisms of the pleural environment-induced invasive phenotype, we used RNA-seq and flow cytometry to compare gene expression profiles and cellular composition of IPL and SC AB1 and AE17 tumors (Figure 2A).

Although CIBERSORT identified some significant differences in cellular content between SC and IPL tumors within either AB1 or AE17, such as an increase of B cells in IPL tumors in the AB1 model, none of these differences were consistent between the models (Figures 2B,S2A, and S2B). As flow cytometry analysis showed an increased number of B cells in IPL tumors in both models (Figure 2C), we tested whether adaptive immune cells, including B cells, were causal or likely epiphenomenal to the difference in tumor size. We inoculated human mesothelioma cell line VGE62 either IPL or SC in NOD.Cg-Prkdc^scid^ Il2rg^tm1WjI^/SzJ (NSG) mice, which lack functional B, T, and NK cells (Maletzki et al., 2019). Pleural tumors again had an increased mass, compared to SC, demonstrating that the difference was not related to the adaptive immune system (Figure 2D).

Next, we performed differential gene expression analysis (Love et al., 2014), which identified 419 genes differentially expressed between IPL and SC tumors (Figure 2E). Of these, 144 were underexpressed and 275 were overexpressed in IPL tumors, compared to SC. Unsupervised hierarchical clustering was used to visualize the relative expression of these genes across samples, which indicated a clear separation of invasive and non-invasive tumors (Figure 2F). To identify the factors predicted to drive the observed transcriptional response in IPL tumors, common to both models, we used Ingenuity Pathway Analysis (Krämer et al., 2014) on the 419 intersected differentially expressed genes. Three of the top ten most significant predicted upstream transcription factors relate to peroxisome proliferator-activated receptor (PPAR) signaling, namely PPARα, PPARγ, and retinoid X receptor α (RXRα) (Figure 2G).

To map the biological patterns underlying the differences between IPL and SC tumors, we used weighted gene correlation network analysis (WGCNA) (Langfelder and Horvath, 2008). This identified four modules that were significantly upregulated in IPL tumors (p <0.01) in both tumor models (Figures S2C and S2D). Interestingly, one of these, the turquoise module, showed *Ppara* and *Pparg* in the top 25 predicted upstream regulators, and the assembled network in Cytoscape (Lopes et al., 2010) identified *Pparg* as a hub (Figure 2H). PPARγ has been shown to drive invasive behavior of tumor cells by increasing cell motility in other cancer types (Nakajima et al., 2008). These data suggested that PPAR_α/γ_ could coordinate the invasive and proliferative phenotype of pleural mesothelioma.

### Endogenous PPAR ligand arachidonic acid is highly abundant in pleural mesothelioma

**Figure 3:**
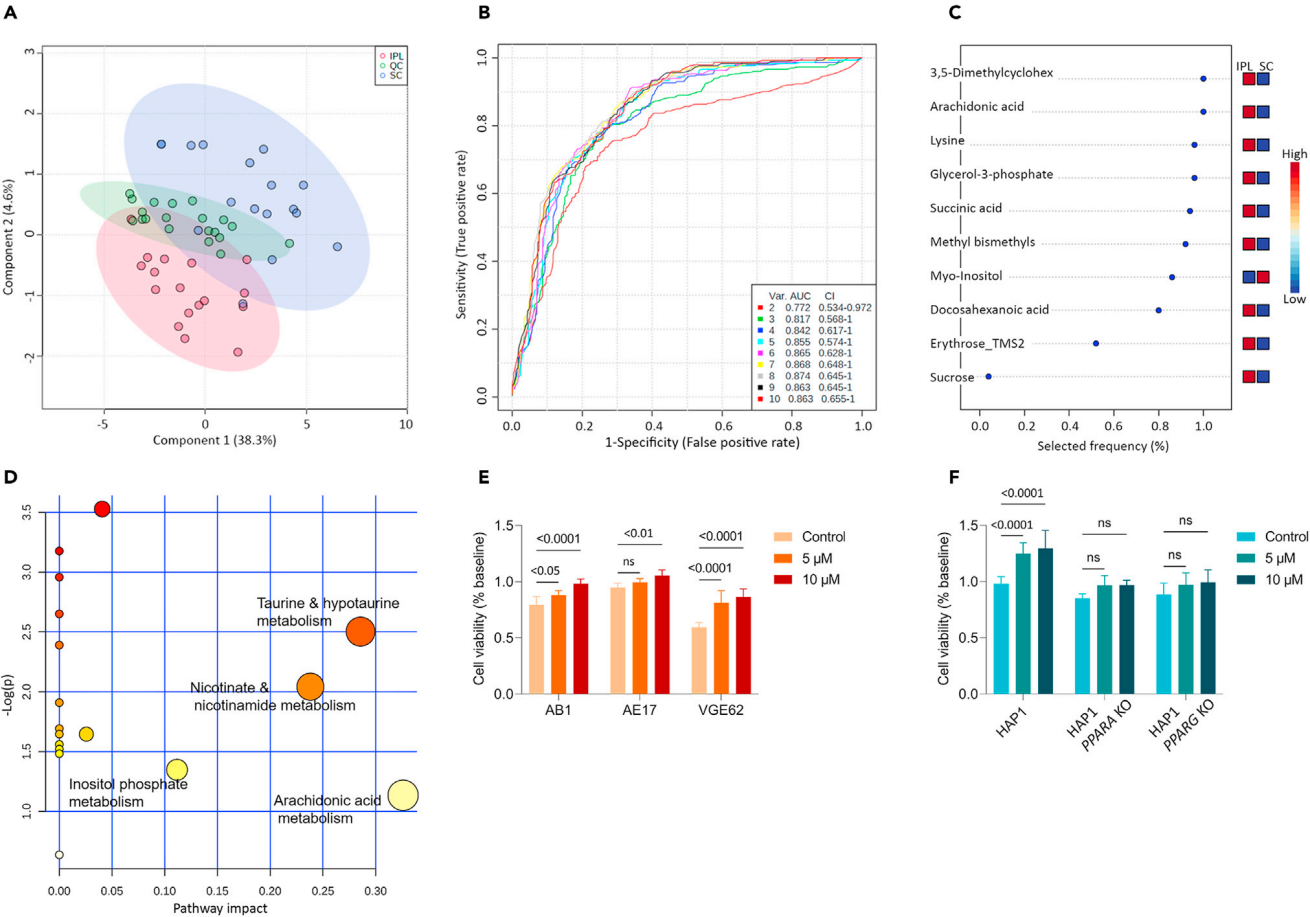
Endogenous PPAR ligand arachidonic acid is highly abundant in pleural mesothelioma (A–D) Tumor inoculation of AE17 and AB1 cells induces mesothelioma tumor tissue growth with a location-specific biochemical fingerprint. (A) Differing between the intrapleural (IPL) (red) or subcutaneous (SC) (blue) implantation location, as observed by PLS-DA. (B and C) The 10 most influential metabolites which describe the observed biochemical phenotypes, modeled by ROC Curve analysis (B) ROC curves for individual biomarker models based on the average model performance, using between two and ten of the identified metabolites (C) The most influential metabolites ranked by importance. (D) Metabolomic pathways analyses. Significant pathways are displayed as circles and the color of each circle is displayed as p value (Y axis). The size of the circle corresponds to the pathway impact score (X axis). (E and F) Cell metabolic activity assay using MTT in (E) AB1, AE17, VGE62 (F) HAP1, HAP1 *PPARA* KO, and HAP1 *PPARG* KO cell lines. Arachidonic acid was used at 0 (control), 5, and 10 μM. Data are depicted as means ± SD values of three independent experiments, n (replicates per experiment) = 3 per group. Two-way ANOVA with Dunnett's multiple comparison test.

As PPARα and γ are ligand-activated transcription factors involved in the regulation of glucose and fat metabolism (Lehrke and Lazar, 2005), we analyzed the metabolite profiles of the pleural and subcutaneous tumors in both AB1 and AE17 cell lines using gas chromatography/quadrupole time-of-flight mass spectrometry (GC/Q-TOF). Unsupervised multivariate data modeling using principal component analysis (PCA) identified the cell lines themselves as significant contributors to the variance between metabolite profiles (Figure S3A). PCA also revealed metabolic differences between invasive and non-invasive tumors. The differences between these metabolomes were further explored using partial least squares discriminant analysis (PLS-DA), which defined a clear separation between the IPL and the SC groups, including analytical quality controls (Figure 3A). Subsequently, receiver operating characteristic curve analysis (ROC) was used to determine the most influential metabolites, defining the invasive versus non-invasive phenotypes (Figures 3B and 3C). Most notable among these markers were myo-inositol, more abundant in the non-invasive subcutaneous tissue (SC), and the lipids arachidonic acid and another long-chain fatty acid identified as docosahexaenoic acid, which were more abundant in the invasive pleural tumors (IPL).

To understand the impact of the differentially activated metabolic pathways, a topology analysis using MetaboAnalyst 4.0 (Xia et al., 2015) was performed. Arachidonic acid metabolism displayed the greatest impact value (>0.30) when compared with other significant metabolites (Figure 3D). Strikingly, arachidonic acid is a known PPARα- and γ-activating ligand (Fang et al., 2007), which is released after injury or irritation as an inflammatory mediator (Tavolari et al., 2008), and has been previously linked to cancer progression (Avis et al., 2005; Chang et al., 2013). These data indicate that arachidonic acid is abundant in pleural tumors.

Based on these findings, we then evaluated if arachidonic acid increased proliferation in murine mesothelioma cell lines AB1 and AE17, and the human mesothelioma cell line VGE62. We observed increased proliferation in a concentration-dependent manner (Figure 3E; p. value <0.05). To test whether this effect was PPAR-dependent, we measured proliferation of the haploid human leukemia cell line HAP1, and genetically engineered versions that lack functional proteins PPARα (HAP1 *PPARA* KO) or PPARγ (HAP1 *PPARG* KO), after adding arachidonic acid. We observed a significant increase in proliferation, but only in the HAP1 parental cell line, not in the *PPARA*/*PPARG* KO cells (Figure 3F; p. value <0.0001), suggesting that arachidonic acid induced increased mesothelioma cell proliferation in a PPARα- and PPARγ-dependent manner.

### Chemical probe GW6471 is a dual PPARα/γ antagonist

**Figure 4:**
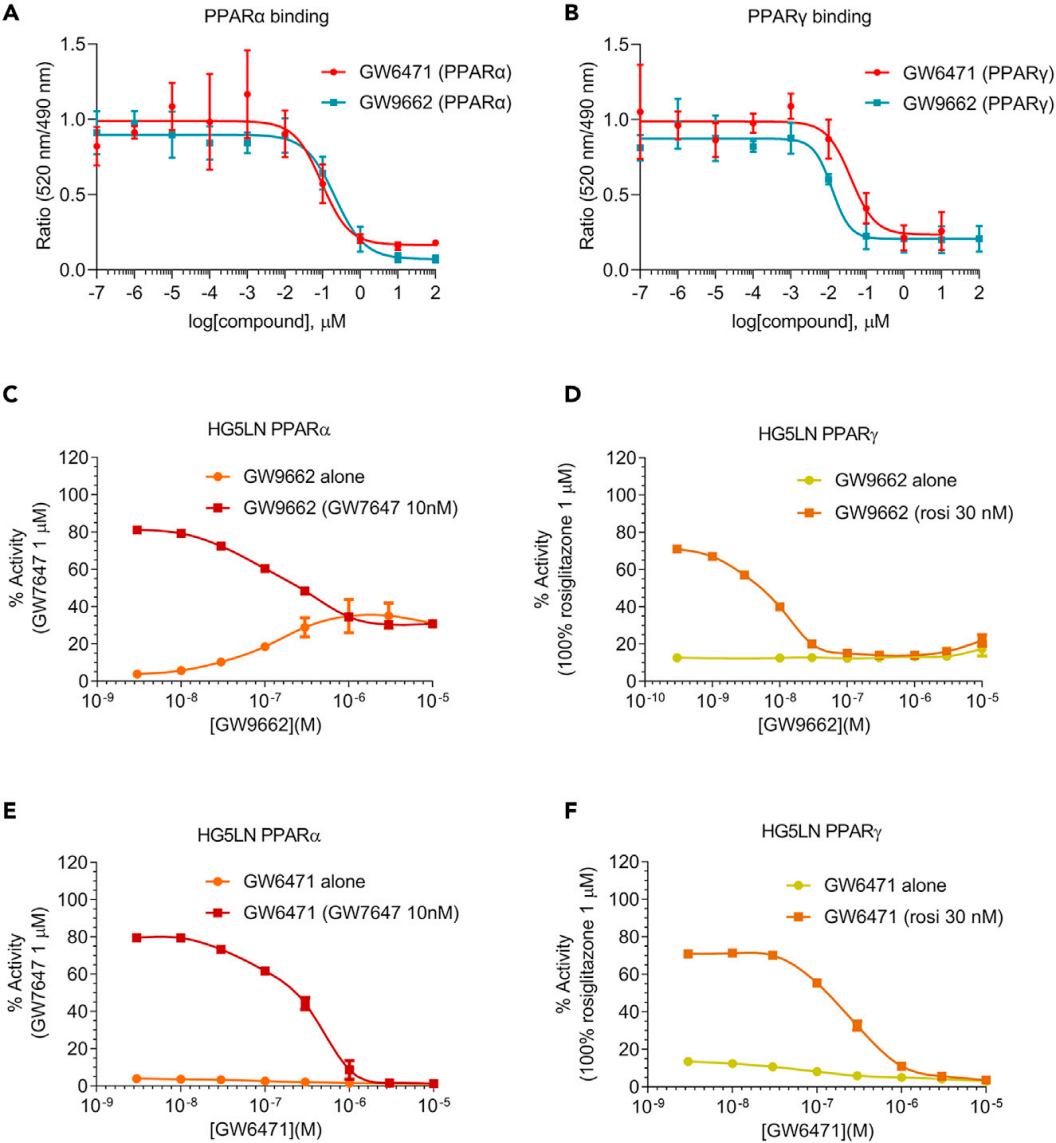
Chemical probe GW6471 is a dual PPARα/γantagonist (A and B) LanthaScreen TR-FRET binding assay for chemical probes GW6471 and GW9662 when binding to (A) PPARα and (B) PPARγ. The curve is presented as a non-linear regression; log(ligand) versus response. IC_50_ values (μM) are shown in Table 1. Data are presented as means ± SD values, n (replicates per experiment) = 3 per group. (C–F) Reporter cell assay using HG5LN for (C) GW9662 vs PPARα (D) GW9662 versus PPARγ (E) GW6471 versus PPARα, and (F) GW6471 versus PPARγ. Data represented as squares indicate the antagonistic activity of the chemical probes in the presence of PPARα agonist GW327647 or the PPARγ agonist rosiglitazone. Data represented as circles are from control experiments to examine the effect of the chemical probes on the viability of the cell line, HG5LN. The curve is presented as a non-linear regression; log(ligand) versus response. IC_50_ values (μM) are shown in Table 1. Data are presented as means ± SD values. n (replicates per experiment) = 4 per group.

Because we identified PPARα and PPARγ as potential regulators of a transcriptional program underlying the invasive and proliferative behavior of mesothelioma in the pleural space, we hypothesized that antagonizing these regulators would alter tumor cell invasion and proliferation. Chemical probes are small molecules that can bind and alter the function of a biological target (Blagg and Workman, 2017). Chemical probes GW6471 and GW9662 were reported (and are marketed) as selective PPARα (Xu et al., 2002) and PPARγ (Leesnitzer et al., 2002) antagonists, respectively. GW9662 is also a known partial agonist of PPARα (Leesnitzer et al., 2002). To confirm the selectivity of these compounds, we used a LanthaScreen™ TR-FRET binding assay. As expected, GW9662 had high affinity for both PPARα and γ. Surprisingly, however, GW6471 also exhibited high affinity for PPARγ (Figures 4A, 4B, and Table 1).

**Table 1:** LanthaScreen™ TR-FRET binding and reporter cell HG5LN assay for PPARα- and γ-mediated transcription using chemical probes GW9662 and GW6471. Reference IC_50_ values of GW6471 and GW9662 for PPARα and PPARγ are also shown.

					Functionality		
	Compound	Binding assay, IC_50_ (nM)^a^	Lit. Binding assay, IC_50_ (nM)	Reporter cell assay, IC_50_ (nM)^e^	Type	Lit. IC_50_ (nM)	Lit. EC_50_ (nM)
PPARα	GW6471	95.3 ± 42.0	–	254 ± 96	antagonist	240^f^	–
	GW9662	213.9 ± 64.6^b^	39 ± 4^c^	131 ± 31	partial agonist	188 ± 43^g^	26 ± 9^j^
PPARγ	GW6471	39.4 ± 18.2	–	210 ± 64	antagonist	<1000^h^	–
	GW9662	12.4 ± 3.6^b^	5.4 ± 0.6^d^	4 ± 2.6	antagonist	3.8 ± 1.3^i^	–

a Values are the mean of 3 replicates ± SEM.

b NB: 2 h incubation time. GW9662 is an irreversible inhibitor, so measured binding affinities are strongly influenced by incubation time.

c Displacement of [^3^H]GW2331 (Leesnitzer et al., 2002). Incubation time 1 h.

d Displacement of [^3^H]rosiglitazone (Leesnitzer et al., 2002). Incubation time 1 h.

e Values are the mean of 3 experiments with 4 replicates ± SEM. Inhibition of agonism by 10 nM GW7647 (PPARα) and 30 nM rosiglitazone (PPARγ). All compounds were incubated for 16 h.

f Inhibition of agonism by 10 nM GW409544, a structurally related agonist (Xu et al., 2002).

g Inhibition of agonism by 30 nM GW7647 (Seimandi et al., 2005).

h An IC_50_ value was not reported (Goldstein et al., 2017).

i Inhibition of agonism by 20 nM rosiglitazone (BRL49653) (Seimandi et al., 2005).

j 33% of maximal activity (Seimandi et al., 2005).

Nuclear receptors, including PPARα and γ, are ligand-inducible transcription factors that form heterodimers with retinoid X receptors and bind to specific DNA sites located in the enhancer/promoter region of target genes, releasing co-repressors and/or recruiting co-activators (Wang, 2010). We therefore assessed the functional consequences of the ligands on PPARα and γ-mediated transcriptional activation in reporter cell lines. These cell lines were generated as previously reported by a two-step transfection procedure (Seimandi et al., 2005). First, a stable cell line, HG5LN, expressing the reporter gene (GAL4RE-luciferase) was developed from the HeLa cell line. These cells were then transfected with a plasmid expressing the C-terminal ligand-binding domain of PPARα or γ fused to GAL4 DNA-binding domain. The parental HG5LN cells were used as a control to test non-specific modulation of luciferase expression by the small molecules investigated (Figure S4A). The HG5LN PPAR reporter cells therefore allow comparison of the functional activity at, and selectivity of different ligands for, PPARα and γ. As reported previously (Seimandi et al., 2005), reporter cell lines assays showed that GW9662 fully antagonizes PPARγ (Figure 4D and Table 1), and partially agonizes PPARα (Figure 4C and Table 1) at nanomolar concentrations. As expected (Abu Aboud et al., 2015), GW6471 potently antagonized PPARα (Figure 4E and Table 1). Here, in addition and in-line with the binding affinity assay, we show that it is also a PPARγ antagonist of similar potency (Figure 4F and Table 1). We then tested GW6471 on PPARδ, which showed no effect (Figure S4B). A recent study reported GW6471 as a modest PPARγ antagonist (Goldstein et al., 2017), while previous studies only showed PPARα antagonism (Abu Aboud et al., 2015; Gaspar et al., 2020), and while GW6471 is marketed as a PPARα-selective antagonist. Our data show that GW6471 is actually a potent, dual PPARα/γ antagonist.

### Dual PPARα/γ antagonist GW6471 inhibits cancer cell growth and migration *in vitro*

**Figure 5:**
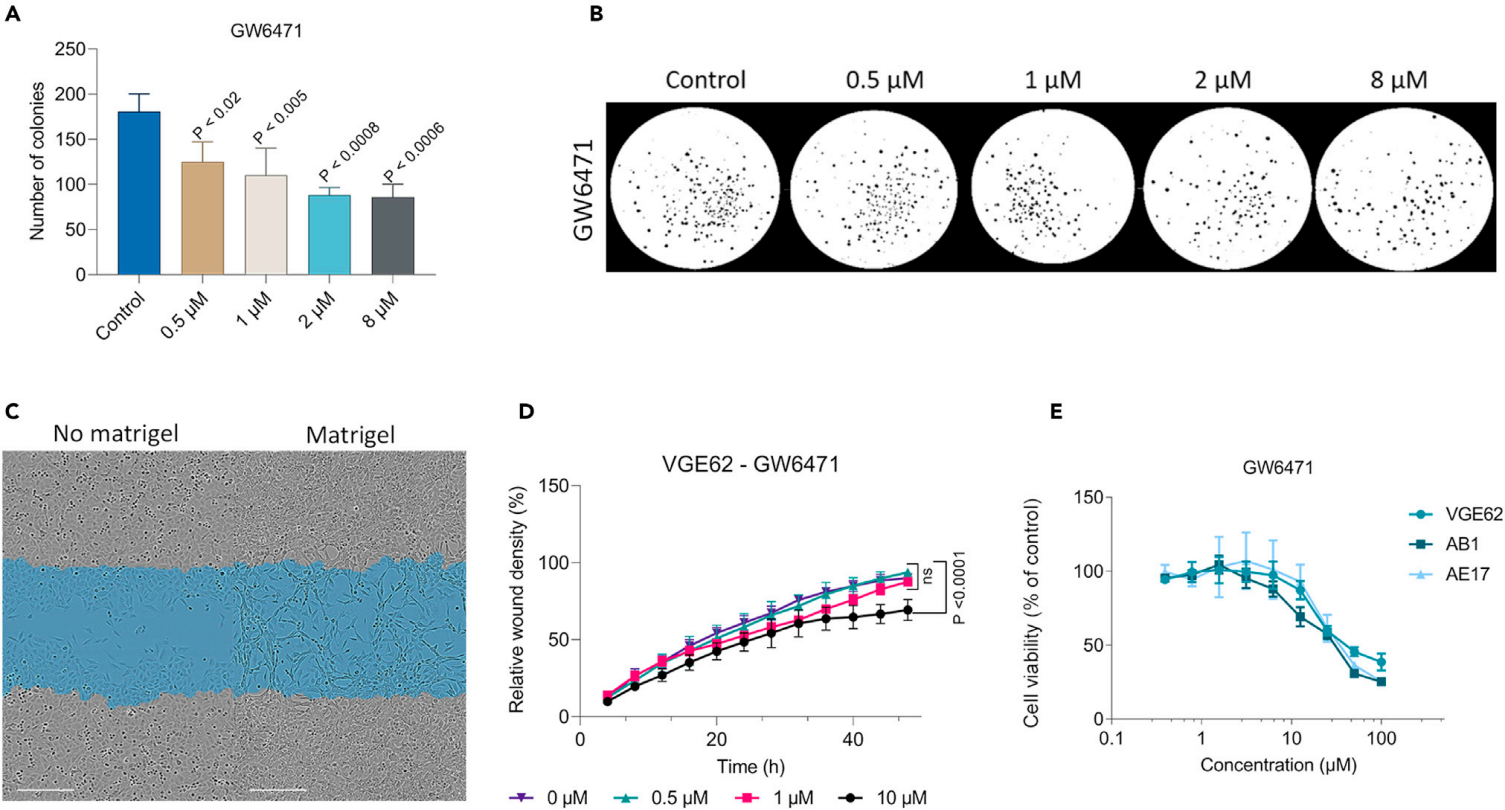
Dual PPARα/γ antagonist GW6471 inhibits cancer cell growth and migration *in vitro* (A and B) Soft agar colony formation assay for JU77 human mesothelioma cell line. GW6471 was added in different concentrations, 8, 2, 1, 0.5 μM, and the control (0 μM). (A) Data are means ± SD of three replicates. Two-way ANOVA with Dunnett's multiple comparison test (∗p <0.02, ∗∗p <0.005, ∗∗∗p <0.001). (B) Colonies count was performed using the software ImageJ. Edges were excluded, size = pixel^2^ 0 – infinity, circularity = 0.00–1.00. (C) Scratch assay for migration (control, no matrigel) and invasion (matrigel) in wounded VGE62 human mesothelioma cell line. Original wound in blue. Scale bar = 300 μm. (D) Invasion assay in wounded VGE62 human mesothelioma cell line when adding GW6471 at 10, 1, 0.5 μM, and control (0 μM). Data are means ± SD values of three replicates, n (replicates per experiment) = 4. Two-way ANOVA with Dunnett's multiple comparison test. (E) Cell metabolic activity assay using MTT in VGE62, AB1, and AE17 cell lines. GW6471 was serial diluted from 100 μM to 0.391 μM and a control (0 μM). Cell viability was normalized (% of control). Data are means ± SD values of three replicates, n (replicates per experiment) = 4.

Having confirmed that GW6471 effectively antagonizes both PPAR α and γ, we tested whether this compound affects proliferation using soft agar colony formation assays with the human mesothelioma cell line JU77. We found a significant reduction in the number of colonies formed when JU77 cells were cultured in the presence of GW6471 (p. value <0.05) (Figures 5A and 5B).

We then evaluated the ability of human mesothelioma cell line VGE62 to invade into matrigel, as a surrogate for the extracellular matrix, and observed that horizontal growth was comparable with and without matrigel (Figure 5C). Additionally, when we measured the relative wound density, which is the ratio of the area occupied by the cells to the total area of the initial scratched region, GW6471 significantly inhibited invasion in this assay, in a concentration-dependent manner (Figure 5D; p. value <0.0001), at concentrations that did not significantly affect cell proliferation, indicating that the inhibitory effect on invasion was not due to cytotoxicity (Figure 5E). In addition to reducing *in vitro* invasion, GW6471 also lowered viability of VGE62 and murine mesothelioma cell lines AB1 and AE17 (Figure 5E), at higher micromolar concentrations.

### Dual inhibition of PPARα/γ does not result in significant anti-mesothelioma activity *in vivo*

**Figure 6:**
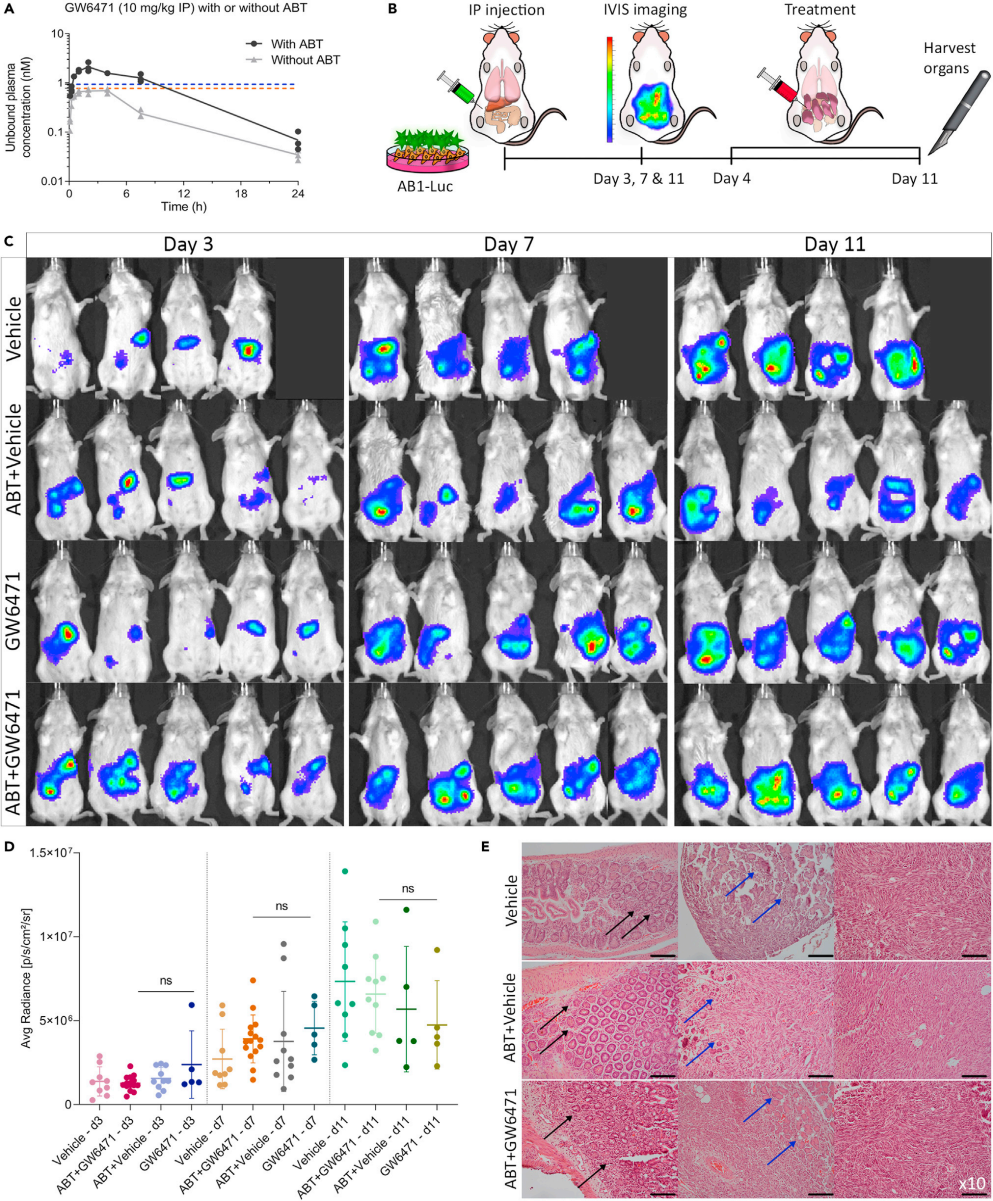
Dual inhibition of PPARα and PPARγ does not result in significant anti-mesothelioma activity *in vivo* (A) Unbound plasma concentration (nM) time profile for GW6471 following IP administration at 10 mg/kg in female BALB/c mice with and without pre-treatment with ABT. Dotted lines show *in vitro* reporter cell assay IC_50_ values (corrected for unbound fraction) for PPARα in blue and PPARγ in orange. Data are presented as individual dots, n (mice per group) = 3. (B) Experimental design. (C) Bioluminescence imaging for tumor bioluminescence monitoring on days 3, 7, and 11 for control groups (vehicle and ABT + vehicle) and treatment groups (GW6471 and ABT + GW6471). (D) Tumor bioluminescence comparison based on average radiance (p/s/cm^2^/sr) over time. Data are means ± SD values of two replicates, n = 5. Two-way ANOVA with Dunnett's multiple comparison test. (E) IP tumors were stained with hematoxylin and eosin. Tumor invasion in the intestines is indicated by black arrows and in the pancreas by blue arrows. Scale bar = (x10) 100 μm.

The identification of GW6471 as the first potent, dual PPARα/γ antagonist (Figures 4E and 4F) provided the opportunity to test the hypothesis that concurrent antagonism of both PPARα and γ could be an effective mesothelioma treatment. Before proceeding to an *in vivo* mesothelioma model, we tested whether the concentration required for full target inhibition, as identified in the transcriptional reporter assays, could be achieved *in vivo*. This was assessed in female BALB/c mice by monitoring total plasma concentrations of GW6471 following IP administration at 10 mg/kg. As *in vitro* metabolic stability studies suggested that GW6471 is rapidly metabolized by cytochrome P450 (CYP) enzymes (degradation half-life of 3 min and *in vitro* CL_int_ of 556 μL/min/mg protein in NADPH-activated mouse liver microsomes, but stable in the absence of NADPH), one group of mice was treated with the pan-CYP inhibitor, 1-aminobenzotriazole (ABT), prior to GW6471 dosing, with the goal of increasing plasma exposure levels to achieve consistent full target (PPAR) inhibition. Following IP administration of GW6471, there were measurable plasma concentrations over the 24-h sampling period for both animal groups and the plasma concentration of GW6471 was approximately 3-fold higher with ABT pre-treatment (based on both area under the curve (AUC) and C_max_). In order to account for differences in plasma protein binding when comparing the plasma concentrations of GW6471 observed *in vivo* with the media concentrations found to be active in the *in vitro* assays, we also measured the unbound fraction for GW6471 in both plasma and reporter cell assay media (Supplementary Table S1) and then based the comparison on the unbound concentrations (i.e. the pharmacologically achieved concentration). Plasma concentration-time profiles of GW6471 thus corrected for unbound fraction are shown in Figure 6A, and plasma exposure parameters are summarized in Table 2. Importantly, the unbound concentrations present *in vivo* after dosing GW6471 at 10 mg/kg would be expected to result in full target inhibition (based on the transcriptional reporter assays), but only when mice were co-treated with ABT (Figures 4E and 4F).

**Table 2:** Plasma exposure parameters for GW6471 in BALB/c mice following IP administration in the absence or presence of ABT at 50 mg/kg (GW6471 and ABT + GW6471, respectively).

Parameter	GW6471	ABT + GW6471
Average dose (mg/kg)	10.9	10.4
Plasma AUC_0–24 h_(h∗μM)	9.81	31.4
Plasma C_max_ (μM)	1.18	3.60

We then optimized a model of invasive mesothelioma that would allow assessment of therapeutic efficacy. As the IPL model does not allow serial determination of tumor size, we inoculated BALB/c mice intraperitoneally (IP) with 5 × 10^5^ AB1 cells expressing luciferase (AB1-Luc) and used bioluminescence imaging to track tumor growth. This resulted in reproducible mesothelioma growth that could be quantified *in vivo* over time (Figures S5A and S5B).

Lastly, to assess the contribution of PPARα and γ on cancer cell growth and invasion *in vivo*, GW6471 was administered daily, with or without ABT pre-treatment. Bioluminescence imaging was performed at three time points after inoculation: on day 3 (24 h before treatment), and on day 7 and 11 (during treatment, Figure 6B). These time points were selected based on optimization experiments of the IP model, which demonstrated that after day 11 the overall wellbeing of untreated mice declined.

There was no significant effect on tumor growth in either the GW6471 or the ABT + GW6471 groups when compared with controls (Figures 6C and 6D). To determine whether GW6471 treatment altered cancer invasion, the tumors were analyzed histologically. All the mice, including those in the control groups, showed invasion into the surrounding tissues such as the intestine and pancreas (Figure 6E). Additionally, we stained GW6471 and vehicle-treated tumors for angiopoietin-like 4 (Angptl4), which has been reported to be downstream of activation or inhibition of PPARα and PPARγ (La Paglia et al., 2017). We observed decreased staining of Angptl4 following treatment (Figure S6A), suggesting that PPARα/γ were inhibited by GW6471 *in vivo*. Together, these findings show that therapeutic antagonism of PPARα and γ using GW6471 has no impact on mesothelioma invasion and proliferation *in vivo*.

### The *in vitro* anti-tumor effects of GW6471 are largely off-target

**Figure 7:**
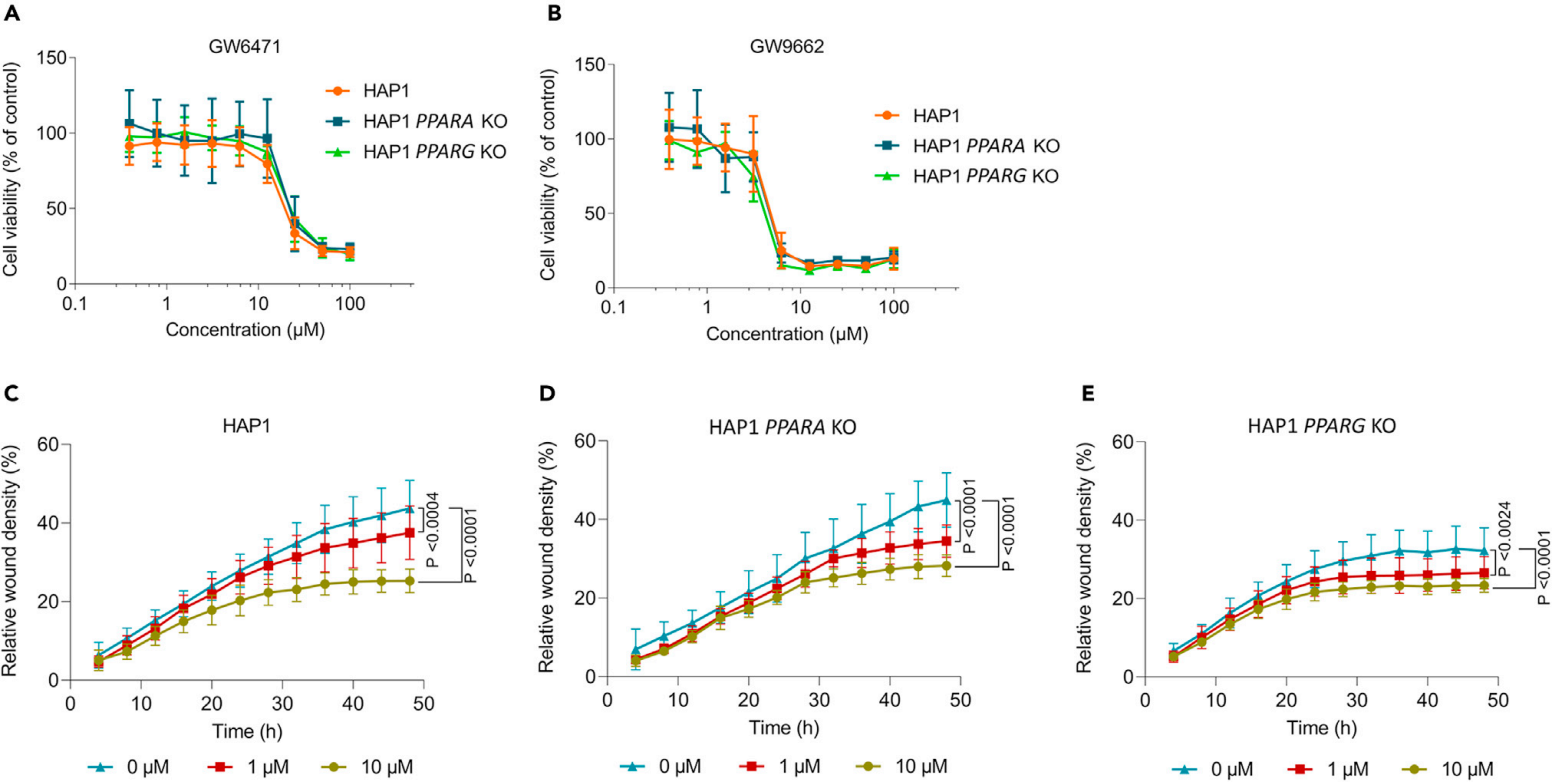
The anti-tumor effects of GW6471 are largely off-target (A and B) Cell metabolic activity assay using MTT in HAP1, HAP1 *PPARA* KO, and HAP1 *PPARG* KO cell lines. (A) GW6471 and (B) GW9662 were serial diluted from 100 μM to 0.391 μM and compared with a control (0 μM). Cell viability was normalized (% of control). Data are means ± SD values of three different experiments with n (replicates per experiment) = 4. (C, D and E) Migration assay in wounded HAP1, HAP1 *PPARA* KO, and HAP1 *PPARG* KO cell lines when adding GW6471 at 10, 1 μM, and control (0 μM). Data are means ± SD values of three different experiments with n (replicates per experiment) = 4. Two-way ANOVA with Dunnett's multiple comparison test. GW6471 significant effect on (C) HAP1; p <0.004 at 1 μM, and p <0.0001 at 10 μM (D) HAP1 *PPARA* KO; p <0.0001 at 1 and 10 μM, and (E) HAP1 *PPARG* KO; p <0.0024 at 1 μM, and p <0.0001 at 10 μM.

Given the negative *in vivo* results, we questioned whether the *in vitro* anti-cancer effect of GW6471 observed here, and by others (Abu Aboud et al., 2013), was on-target. To test this, the HAP1, HAP1 *PPARA* KO, and HAP1 *PPARG* KO cell lines were treated with increasing concentrations of GW6471 and GW9662. MTT assays showed that GW6471 inhibited cellular proliferation at micromolar concentrations, as observed before in the mesothelioma cell lines (Figures 5E and 7A). However, the level of growth inhibition was identical between cells in which PPARα or PPARγ is present or absent (Figure 7A). Similar results were found for GW9662 (Figure 7B).

Lastly, we evaluated the migratory capacity of the HAP1 cell lines, in the presence or absence of GW6471. Again, although GW6471 had a concentration-dependent effect on *in vitro* migration, this was not significantly different between the *PPARA* knockout, *PPARG* knockout, or wild-type cell lines (Figures 7C–7E). Of note, when comparing the background migratory capacity of the three different cell lines, we observed that genetic deletion of *PPARG* reduced their migratory capacity *in vitro* (Figures 7C–7E and S7A). However, we cannot rule out an off-target CRISPR effect in this particular clonal cell line. Together these results suggest that much of the anti-cancer effects of the PPAR chemical probes GW9662 and GW6471 that we observed are likely off-target.

## Discussion

Many different murine models of mesothelioma have been evaluated to study the disease, using subcutaneous or orthotopic transplantation, asbestos exposure, or genetic engineering approaches (Robinson et al., 2014). Each model has its limitations and strengths. Although subcutaneous transplantation models are most widely used, intrapleural transplantation most closely mimics the clinical progression of the disease in terms of morphology, histological features, and tumor growth, and it maintains the relevant anatomic location. It was first described using xenogeneic transplantation into an athymic nude mouse (Colt et al., 1996). Intrapleural transplantation has been achieved both by surgery (Colin et al., 2018) and by direct injection of the cancer cells without surgical procedure (Nagamachi et al., 1998). These models can develop vascular tumor nodules, metastases, local invasion, and pleural effusions, analogous to human pleural cancers (Servais et al., 2011).

In the present study, we aimed to investigate the molecular mechanisms underlying the microenvironmental control of mesothelioma growth and invasion by comparing invasive IPL tumors with non-invasive SC cancer. There are serious challenges associated with intrapleural cell implantation, such as animal wellbeing, post-procedural morbidity, and researcher experience, which have to be considered. For this reason, we also tested whether intraperitoneal injection would give comparable mesothelioma growth and invasion. Broadly, we found that intraperitoneal disease progressed as rapidly as intrapleural disease with a similarly invasive phenotype.

Using two intrapleural models, we identified PPARα and PPARγ as potential regulators of an invasion-associated gene expression signature in mesothelioma. Each PPAR subtype has unique functions that are related to tissue distribution, ligand response, and biochemical properties (Ahmadian et al., 2013). Moreover, PPARs have a controversial role in cancer (Tachibana et al., 2008), with studies showing that PPAR α or γ activation can either promote or inhibit tumor progression (Peters et al., 2012).

We also found statistically differential levels of lipids and other primary metabolites in the different anatomic locations. Interestingly, arachidonic acid, a known PPAR ligand, was more abundant in the pleural space, which is consistent with our findings of PPAR α/γ activation in the transcriptomic data from the same tumor models.

For these reasons, we sought to inhibit both PPARα and γ, hypothesizing it could inhibit tumor invasion and proliferation. GW6471 is an antagonistic chemical probe that was identified through modification of the PPARα agonist GW409544. Its potent agonist activity at PPARα was previously shown by inhibition of GW409544, a structurally related agonist, in cell-based assays (Xu et al., 2002). We performed in-depth studies on the properties of GW6471, including binding assays, cellular transcriptional reporter assays, *in vitro* metabolic stability, and pharmacokinetic analysis. These studies showed that GW6471 is a potent antagonist for both PPARα and γ, with no effect on PPARδ. Intraperitoneal administration of this compound resulted in sufficient biological exposure levels of the drug, when mice were co-treated with the pan-CYP inhibitor ABT. Together, these data demonstrate that GW6471 is a dual PPARα/γ antagonist and that it can be effectively used *in vitro* and *in vivo*.

Others have used GW6471 in cancer studies; 25 μM induced significant cell-cycle arrest and apoptosis in a model of renal cell carcinoma, and significant tumor reduction at 20 mg/kg *in vivo* (Abu Aboud et al., 2015). Additionally, in an *in vitro* head and neck paraganglioma study, GW6471 reduced cell viability and growth by cell-cycle arrest and caspase-dependent apoptosis at 24 μM (Florio et al., 2017).

In our mesothelioma model, using a low concentration of GW6471, we obtained exposure levels that are consistent with full PPARα/γ inhibition *in vivo*, but we did not find any effect on invasion or proliferation. Although we obtained clear concentration-dependent inhibition of tumor colony formation using low micromolar concentrations of GW6471, it was noted that the IC_50_ for cell growth was several orders of magnitude greater than the affinity and functionality IC_50_ values determined (Table 1). Moreover, we found identical results in PPARγ or α deficient cell lines, demonstrating that the *in vitro* anti-cancer effects of GW6471 at these concentrations are likely off-target, although we cannot fully exclude differential GW6471 effects between tissues *in vivo* and the HAP1 cell line *in vitro*.

These results should caution others against the use of GW6471 as a PPARα (and especially PPARα-specific) antagonist because any observed effect of GW6471 may also be caused by inhibition of PPARγ or a combination of both. In addition, the poor metabolic stability of GW6471, and high plasma protein binding, calls into question any *in vivo* studies that attribute biological activity of this chemical probe to PPARα antagonism.

Although our results show that dual targeting of PPARα/γ is not an effective treatment strategy for mesothelioma, we note that genetic deletion of PPARγ may reduce invasion and proliferation of tumors *in vitro*. Interestingly, a recent study in brain cancer reported the effect of the PPARγ inverse agonist T0070907, which is structurally very similar to GW9662 (Zou et al., 2019). The authors found a significant decrease of brain metastases from melanoma and breast cancer in murine models, but did not find an inhibitory effect on tumor growth in a SC model of melanoma or a metastatic model of lung cancer, underscoring the importance of the tumor microenvironment when targeting this transcription factor. Similarly, Yang *et al*. recently showed that targeting the interaction between PPARγ and nuclear receptor Nur77 results in anti-cancer activity in several mouse models of breast cancer (Yang et al., 2020). These data suggest that targeting PPARγ alone could still form an effective therapeutic avenue in the context of cancer.

Altogether, our data invalidate dual antagonism of PPARα and γ to reduce the growth or invasion of mesothelioma. However, they do provide a resource to investigate the biology associated with the location-dependent phenotype of mesothelioma.

### Limitations of the study

Our studies used RNA-seq of whole tumor tissue. To determine the source of differences (from the tumor cells or from stromal of infiltrating cells), we would need to analyze single cells or sorted tumor cells. Our *in vitro* experiments were performed in *PPARA* or *PPARG* single knockout HAP1 cell lines and we used the dual PPARα/γ antagonist GW6471; we did not genetically delete both these genes in our cellular systems. Additionally, we inhibited PPARα and γ signaling simultaneously using GW6471, but it is possible that selective antagonism of PPARγ, as per other publications (Masuda et al., 2005; Goldstein et al., 2017) could still be effective.

## STAR★Methods

### Key resources table

**Table:** 

REAGENT or RESOURCE	SOURCE	IDENTIFIER
**Antibodies**		

GAPDH (14C10) Rabbit mAb	Cell Signaling	Cat#2118; RRID: AB_10693448
PPARγ (D69) Antibody	Cell Signaling	Cat#2430; RRID:AB_823599
Anti-rabbit IgG, HRP-linked Antibody	Cell Signaling	Cat#7074; RRID:AB_2099233
ANGPTL4 Polyclonal Antibody	Invitrogen	Cat#409800; RRID:AB_2533491
biotinylated goat anti-rabbit IgG	Vector Laboratories (Kit component)	Cat#PK-4001
See Table S2 for flow cytometry antibodies		N/A

**Biological Samples**		

CD1 Mouse Liver Microsomes	SEKISUI XenoTech	Cat#M1000 Lot#1510256

**Chemicals, Peptides, and Recombinant Proteins**		

1-aminobenzotriazole (ABT)	Sigma-Aldrich	Cat#A3940
GW6471	Cayman Chemical	Cat#11697 CAS#880635-03-0
Kollisolv PEG E 400	Sigma-Aldrich	Cat#06855
Kolliphor HS-15 (Solutol)	Sigma-Aldrich	Cat#42966
GW9662	Cayman Chemical	Cat#70785 CAS#22978-25-2

**Critical Commercial Assays**		

LanthaScreen™ TR-FRET PPAR gamma Competitive Binding Assay Kit, goat	ThermoFisher Scientific	Cat#PV4894
eBioscience™ Foxp3/Transcription Factor Staining Buffer Set	ThermoFisher Scientific	Cat#00-5523-00
Vectastain® ABC kit, peroxidase (Rabbit IgG)	Vector Laboratories	Cat#PK-4001
DAB Substrate Kit, Peroxidase (HRP), with Nickel, (3,3′-diaminobenzidine)	Vector Laboratories	Cat#SK-4100
Tumor dissociation kit, mouse	Miltenyi Biotec	Cat#130-096-730

**Deposited Data**		

RNA-seq	This paper	GEO: GSE180618

**Experimental Models: Cell Lines**		

JU77[human mesothelioma cell line]	(Manning et al., 1991)	RRID:CVCL_2536
Line-1 [mouse lung cancer cell line]	Gift from Dr John G. Frelinger (McLean et al., 2004)	N/A
HAP1 parental [human leukemia cell line]	Horizon Discovery	Cat#C631; RRID:CVCL_Y019
HAP1 PPARA KO [human leukemia cell line]	Horizon Discovery	Cat#HZGHC002330c003; RRID:CVCL_TF25
HAP1 PPARG KO [human leukemia cell line]	Horizon Discovery	Cat#HZGHC5468; RRID:CVCL_XR75
AE17 [mouse mesothelioma cell line]	CellBank Australia	Cat#CBA-0156; RRID:CVCL_4408
AB1 [mouse mesothelioma cell line]	CellBank Australia	Cat#CBA-0144; RRID:CVCL_4403
AB1-Luc [mouse mesothelioma cell line]	(Fear et al., 2019)	N/A
HG5LN [HeLa reporter cell line]	Balaguer et al. (2001), Seimandi et al. (2005)	N/A
VGE62 [human mesothelioma cell line]	(Manning et al., 1991)	RRID:CVCL_W084

**Experimental Models: Organisms/Strains**		

Mouse: C57BL/6JArc	Animal Resource Centre (Murdoch, WA, Australia)	Product code: B6 JAX stock number: 000664
Mouse: BALB/cArc	Animal Resource Centre (Murdoch, WA, Australia)	Product code: BC
Mouse: BALB/c	Monash Animal Research Platform (MARP)	N/A

**Oligonucleotides**		

Primers for HAP1 cell lines PPARA and PPARG confirmation	This paper	STAR Methods

**Software and Algorithms**		

See Table S3 for software and algorithms		N/A

### Resource availability

#### Lead contact

Further information and requests for resources or reagents should be directed to and will be fulfilled by the lead contact, Joost Lesterhuis (willem.lesterhuis@uwa.edu.au).

#### Materials availability

This study did not generate new unique reagents.

#### Data code and availability

The generated datasets used in this manuscript have been deposited at Gene Expression Omnibus (GEO) and are publicly available as of the date of publication. Accession number is GSE180618.

This paper does not report original code.

Any additional information required to reanalyse the data reported in this paper is available from the lead contact upon request.

### Experimental model and subject details

#### Cell culture

Cell lines AE17 (Jackaman et al., 2003) and AB1 (Davis et al., 1992) were obtained from and verified by CellBank Australia. AB1 was transfected to express the luciferase (AB1-Luc) (Fear et al., 2019). Cell lines VGE62, JU77 were established in-house by the method of Manning *et al*. (Manning et al., 1991). Cell line Line-1 was a gift from Dr John G. Frelinger (University of Rochester, School of Medicine and Dentistry, Rochester, New York) (McLean et al., 2004). Cell lines HAP1, HAP1 *PPARA* KO, and HAP1 *PPARG* KO were obtained from Horizon. CRISPR/Cas9 methodology was used by Horizon to prepare both *PPARA* and *PPARG* knockouts. Confirmation of *PPARA* function loss and *PPARG* deletion was done with Sanger sequencing by the Australian Genome Research Facility. AB1, AE17, VGE62, and JU77 were maintained in complete R10 medium, RPMI 1640 (Invitrogen) supplemented with 10% foetal calf serum (FCS; Life Technologies), 20 mM HEPES (Sigma-Aldrich), 0.05 mM 2-mercaptoethanol (pH 7.2; Merck, Kilsyth, Australia), 60 μg/mL penicillin (Life Technologies), 50 μg/mL gentamicin (David Bull Labs). Line-1 was maintained in DMEM/F12 (Life Technologies) supplemented with 10% FCS, 100 U/mL penicillin (Life Technologies), 50 μg/mL gentamicin (David Bull Labs), sodium pyruvate 100X (1%) (Life Technologies), non-essential amino acids solution 100X (1%) (Life Technologies). HAP1, HAP1 *PPARA* KO, and HAP1 *PPARG* KO were maintained in IMDM (Life Technologies) supplemented with 10% FCS (Life Technologies), 100 U/mL penicillin (LifeTechnologies) and 100 μg/mL streptomycin (Life Technologies). All cell lines were confirmed mycoplasma negative by polymerase chain reaction. All cells were cultured as a monolayer at 37°C in a humidified atmosphere with 5% CO_2_. Cells were passaged at approximately 1:10-1:5 every 2-3 days when reached 75-80% confluence.

#### Mice

This study was conducted in accordance with the institutional guidelines of the Harry Perkins Institute of Medical Research Animal Ethics Committee (approvals AE057 and AE155). Female BALB/c and Female C57BL/6 mice were obtained from the Animal Resource Centre (Murdoch, WA, Australia). All mice were 8–10 weeks of age when used for the experiments. Mice were housed at the Harry Perkins Institute of Medical Research Bioresources Facility under pathogen-free conditions at 21°C– to 22°C with a 12/12 h light cycle. Cages (Tecniplast) had an individual air filtered system and contained aspen chips bedding (TAPVEI). Mice were fed Rat and Mouse cubes (Specialty Feeds) and had access to filtered water.

#### Tumour cell inoculation

Cells were trypsinized and washed two times in phosphate buffer saline (PBS) and counted with trypan blue dye. For the SC tumour model, mice were shaved on the right-hand flank and inoculated with 5x10^5^ AB1 or 1x10^6^ AE17 cells suspended in 100 μL PBS. Tumour volume (mm^3^) was monitored with callipers. For the IPL tumour model, mice were anaesthetised under continuous isofluorane and inoculated with 5 x 10^5^ AB1 or 1 x 10^6^ AE17 cells suspended in 200 μL PBS into the pleural space as previously described (Lansley et al., 2015) Tumour size was determined by weighting together the tumour with the lungs minus the weight of aged matched healthy lungs. For the IP tumour model, mice where inoculated in the intraperitoneal cavity on the right flank with 5x10^5^ AB1-Luc cells suspended in 200 μL PBS. Tumour size was determined by *in vivo* imaging system (IVIS), see below. All mice were euthanized in accordance with animal ethics guidelines.

#### Mouse pharmacokinetic studies

Pharmacokinetic animal studies were conducted using established procedures in accordance with the Australian Code of Practice for the Care and Use of Animals for Scientific Purposes, and the study protocols were reviewed and approved by the Monash Institute of Pharmaceutical Sciences Animal Ethics Committee. The systemic exposure of GW6471 was studied in non-fasted female BALB/c mice (5 - 8 weeks) weighing 15.2–22.6 g. Mice had access to food and water *ad libitum* throughout the pre- and post-dose sampling period. GW6471 (10 mg/kg) was dosed to all mice via a single intraperitoneal (IP) injection (5 mL/kg). For the ABT pre-treated group, ABT was pre-dosed to animals at 50 mg/kg via IP injection (5 mL/kg) 2 h prior to administration of GW6471. Following IP administration of GW6471, blood samples were collected up to 24 h at indicated time points (n = 3 mice per time point) with a maximum of three samples from each mouse. Blood samples were collected via submandibular bleed (approximately 120 μL) into polypropylene Eppendorf tubes containing heparin as anticoagulant and stabilisation cocktail (containing the protease inhibitor cocktail Complete® and KF) to minimise the potential for *ex vivo* compound degradation in blood/plasma samples. Once collected, blood samples were centrifuged immediately, supernatant plasma was removed, and stored at−80°C until analysis by liquid chromatography-mass spectrometry (LC–MS). The plasma concentration versus time profile was defined by the average plasma concentration at each sample time, and PK parameters were calculated using non-compartmental methods (PKSolver Version 2.0).

#### Mouse PPARα and PPARγ inhibition assay

Female BALB/cJAusb mice (10–12 weeks) were inoculated IP with 5 x 10^5^ AB1-Luc on their right flank. Mice were randomly allocated to the different groups on the first treatment day. Treatment with ABT + GW6471 or GW6471 alone started on day 4 after tumour inoculation. IP Injections and concentrations were used as previously described in the “Mouse pharmacokinetic studies” section in experimental model and subject details. Initial mouse weight was measured immediately before the first injection, and it was used to calculate weight change daily. Mice were dosed for a maximum of seven consecutive days. *In vivo* imaging system (IVIS) (see below) was performed on days 3 (before treatment), 7 (during treatment) and 11 (after treatment). Mice were euthanized on day 11 in accordance with animal ethics guidelines and organs were harvested for staining.

#### *In vivo* imaging system (IVIS)

XenoLight D-Luciferin potassium salt (PerkinElmer, VIC, Australia) was used at a 150 mg/kg concentration dissolved in sterile PBS. Approximately 150 μL (15 mg/mL concentration) was injected subcutaneously per mouse. Mice were anaesthetised in a chamber with a controlled flow of isoflurane 2% and oxygen flow rate of 1 L/min. When mice were fully unconscious eye gel was applied to moisturise during the imaging process. Mice were then transferred to the IVIS Lumina II camera chamber and isofluorane was decreased to 0.5–1% and oxygen flow rate to 0.8 L/min. Mice were imaged for 5 and 10 s exposure duration at 13 min post injection.

### Method details

#### CRISPR/Cas9 KOPCRSanger sequencing

The following primers were provided by Horizon, HAP1 *PPARA* KO, PCR size: 354 bp, Forward: *GAGGTTTATGCCTCAGCACTAGAAG*, TM: 57.1°C, Reverse: *ACTGCCATTTGCTATAAAAGTGACC*, TM: 55.8°C. HAP1 *PPARG* KO, PCR size: 292 bp, Forward: *TTTCAGAAATGACCATGGTTGACAC*, TM: 55.9°C, Reverse: *TGAGAGATGAGTCCAATTCTAGTCC*, TM: 55.5°C. PCR setup was performed following manufacturer guidelines on Invitrogen^TM^ Platinum^TM^ II *Taq* Hot-Start DNA Polymerase (14966, Thermo Fisher Scientific Scientific), and the PCR ran for 25 cycles and 60°C as the optimised primer temperature. PCR confirmation was performed with a 1% agarose gel in TAE buffer, with Gel red dye (Invitrogen). Samples were then sent to the Australian Genome Research Facility as unpurified gDNA PCR products to sequence. The resulting files were manually analysed using BLAST® (NCBI) to compare the KO sequences against the parental sequence (Figure S8A).

#### Protein extraction and quantification

Protein extraction with RIPA buffer was performed following the manufacturer guideline's (89900, Thermo Fisher Scientific Scientific). Cells were trypsinised from 6-well plates when ∼80% confluent. RIPA buffer was supplemented with PhosSTOP inhibitor (Merck) at 10X concentration and cOmplete inhibitor (Roche) at 25X concentration. Cells were washed twice with PBS and 200 μL of cold complete RIPA buffer was added to each well and kept on ice for 5 min. The lysate was then collected with a scrapper and centrifuged at maximum speed for 15 min. The supernatant was then collected and quantified with the Pierce BCA Protein Assay Kit (Thermo Fisher Scientific Scientific) as explained by the manufacturer guidelines. The standard curve was prepared in triplicate with albumin standard (BSA) diluted in PBS, working concentration goes from 25 to 2,000 μg/mL within 8 dilutions. The BCA working reagent was prepared by mixing 50 parts of BCA Reagent A with 1 part of BCA reagent B (50:1, Reagent A:B). All the samples were prepared with a 1:8 sample to BCA working reagent ratio in a 96-well plate by replicates, adding 25 μL of each standard or unknown sample and 200 μL of BCA working reagent. The plate was then protected from the light and mixed on a plate shaker for 30 s, following by a 30 min incubation at 37°C. Absorbance was then measured at 562 nm on a plate reader and the standard curve was used to determine the protein concentration of each sample.

#### Western blotting

Protein lysates were thaw and diluted to a final concentration of 30 μg of protein and combined with 4X loading buffer (4X Laemmli Sample Buffer completed with 2-mercaptoethanol, Bio-Rad) for a loading volume of 30 μL. Then, the lysates were incubated for 5 min at 95°C. 10% Mini-Protean TGX Pre-cast Protein Gel (Bio-Rad) were used to load the ladder (2 μL, Precision Plus Protein Dual Colour Standards, Bio-Rad), followed by 30 μL of each samples. 1X Western Running Buffer was added to cover the gel (10X Western Running Buffer (SDS-PAGE), 250 mM Tris, 1.92 M glycine, 1% SDS, pH 8.3). The gel was run for 5 min at 50 V and then the voltage increased to 200 V for 25 min. Protein separation was then confirmed by a ChemiDoc (Bio-Rad) using the ImageLab software.

Using the Trans-blot Turbo (Bio-Rad) for 7 min, the gel was then transferred to a 0.2 μm membrane (Trans-blot Turbo Mini Nitrocellulose Transfer Pack, Bio-Rad).

Once transferred, the membrane was blocked with 5% non-fat milk powder in TBST Buffer (Tris-Buffer Saline with Tween, 20 mM Tris, 500 mM NaCl, and 0.05% Tween 20) at room temperature for 60 min, followed by three washes for 5 min in TBST. The primary antibody, GAPDH (1:1000) (2118S, Cell Signaling), or PPAR gamma (1:1000) (2425S, Cell Signaling) was diluted in 5% BSA in TBST and added to the membrane for an overnight incubation at 4°C. The membrane was then washed three times with TBST and the secondary antibody, Anti-rabbit IgG, HRP-linked Antibody (1:2000) (7074P2, Cell Signaling), was diluted in 5% non-fat milk powder in TBST for 90 min at room temperature, followed by three washes for 5 min with TBST. The membrane was then place in the ChemiDoc and total protein density was observed using the ImageLab software.

To visualise the target protein, Clarity Western ECL Substrate (Bio-Rad) was prepared as per manufacturer guidelines at 1:1 ratio and added on top of the membrane to cover the surface for 5 min at room temperature. ECL excess was drained and the membrane placed in the ChemiDoc to visualise the target protein. Figures S8B and S8C.

#### Hematoxylin and eosin staining

Mouse tissues were fixed with 4% paraformaldehyde for 48 - 72 h and embedded in paraffin. 5 μm sections were cut by microtome (Thermo Fisher Scientific) and placed on Microscope Slides with 20 mm Colourfrost blue (Hurst Scientific). Slides were deparaffinised at room temperature as following; 2 rounds of xylene (3 min each), 2 rounds of 100% ethanol (2 min each), 95% ethanol (1 min), 70% ethanol (1 min), 40% ethanol (1 min), 3 rounds of distilled H_2_O (3 min each). Slides were stained with Mayers hematoxylin (Sigma-Aldrich) for 10 min and rinsed with running tap water, the counterstain was performed with acidified Eosin Y solution (Sigma-Aldrich) (0.5% glacial acetic acid) for 45 s. Dehydration was performed as following; 40% ethanol (30 s), 70% ethanol (30 s), 95% ethanol (30 s), 2 rounds of 100% ethanol (1 min each), 2 rounds of xylene (3 min each). Mounting was performed with Pertex mounting medium (Histolab), and sections were imaged under a light microscope.

#### Tumour preparation for RNA-seq

At day 10 after tumour inoculation, mice were euthanized and the tumours and surrounding tissues were harvested. IPL tumours were weighed together with the lungs and total weight recorded. Later, lungs and surrounding tissues were removed and tumours were submerged in RNAlater (Life Technologies) at 4°C overnight to allow RNAlater to penetrate the tissue. Then, tumours were removed from RNAlater and stored at−–80°C until dissociation with TRIzol (Life Technologies) using a TissueRuptor (QIAGEN). RNA was extracted with chloroform and purified on RNeasy MinElute columns (QIAGEN). Library preparation and sequencing at 50 bp single end reads with Illumina HiSeq standard protocols were performed by Australian Genome Research Facility.

#### Flow cytometry

At day 10 after tumour inoculation, mice were euthanized and the tumours were harvested. Surrounding tissue was removed and tumours were submerged in cold PBS, cut into 1–2 mm pieces with a scalpel blade and dissociated with the tumour dissociation kit, mouse (Miltenyi Biotec) using the hard tumour program in the gentleMACS dissociator (Miltenyi Biotec). Cells were incubated at room temperature for 20 min with Zombie UV live/dead (BioLegend). Antibody cocktails were added as following; lymphoid panel: CD45, CD3, CD4, CD8, FoxP3, CD335, CD19, and myeloid panel: CD45, MHCII, CD11b, CD11c, Ly6G, Ly6C, and CD117 for 30 min at room temperature in the dark. Information on antibodies are summarised in Table S2. Cells were permeabilised and fixed using FoxP3 Fix/Perm buffer kit (Life Technologies) for 15 min at room temperature. Intracellular staining was performed using FoxP3 Fix/Perm buffer for 20 min at room temperature and cells were resuspended in stabilizing fixative until acquisition. Data were recorded on the cytometer FACSCantoII and analysed using FlowJo software.

Cell populations were defined as following: CD4 helper T cells (CD3+CD4+FoxP3-), CD8+ T cells (CD3+CD8+), CD19+, NK cells (CD335 + CD3-), NK T cells (CD335 + CD3+), CD117+, CD11c + MHCII+, granulocytes (Ly6G + Ly6C + CD11b+), monocytes (Ly6G-Ly6C-CD11b+), macrophages (CD11b + MHCII+/Ly6C-Ly6G-), DCs (CD11c + MHCII+). See Figure S9 for gating strategy.

#### Bulk RNA-seq analysis

Raw read libraries were quality assessed using FastQC (Andrews, 2010) (v0.11.3) and mapped to the mouse genome (mm11) at both the transcript and gene level using HISAT2(Kim et al., 2015) (v2.0.4). Gene-level quantitation (counts) of aligned reads was performed using SummerizeOverlaps (Lawrence et al., 2013), and transcript discovery and quantification using Stringtie (Pertea et al., 2015) (v1.3.0) and Ballgown (Frazee et al., 2015). Post-alignment quality control was performed using SAMStat (Lassmann et al., 2011) (v1.5.2).

The CIBERSORT (Newman et al., 2015) algorithm was used to estimate the relative proportions of 25 mouse hematopoietic immune cell types within each sample based on their transcriptomic profiles, using the 511 mouse-gene signature developed by Chen et al. (Chen et al., 2017) as a reference. Transcript level data were prepared for CIBERSORT by first applying library size and gene length normalisation using the Ballgown R package (Frazee et al., 2014) resulting in Fragments Per Kilobase of transcript per Million mapped reads (FKPM). Individual transcripts were then collapsed to gene level data based on the mean FPKM value using the aggregate() function. The data were then filtered to retain genes with an FPKM value > 0.3 in at least 8 samples (being the smallest experimental group size).

Differentially expressed genes were identified between IPL and SC tumours within both AB1 and AE17 models using DESeq2 (Love et al., 2014). p values were adjusted for multiple comparisons using the Benjamini–Hochberg (B-H) method. p<0.05 was considered significant. The WGCNA algorithm (Langfelder and Horvath, 2008) was used to construct a signed network across all AB1 or AE17 samples, and identify clusters (modules) of genes with highly correlated patterns of gene expression. Data were prepared for WGCNA for AB1 and AE17 separately by applying a variance stabilising transformation, followed by filtering to remove low or non-expressed genes (those with a count per million equivalent to a count of 20 per sample were retained) or those missing an official MGI symbol. Finally, variable genes were selected by applying the varianceBasedfilter() function within the DCGL package (Yang et al., 2013) (significance threshold set to 0.01). The union of the resulting sets of genes for AB1 and AE17 (5265 genes in total) was used as input for network construction. Network modules of co-expressed genes identified by WGCNA were tested for enrichment of differentially expressed genes between IPL and SC tumours by plotting the–log_10_ p values derived from the DESeq2 analysis, on a module-by-module basis.

Differentially expressed genes and network modules of interest were analysed within Ingenuity Systems (Krämer et al., 2014) to identify predicted upstream transcriptional regulators, using right-tailed Fisher's exact tests and default settings for other options; p values <0.05 were considered significant. Activation Z-scores were calculated for each regulator by comparing their known effect on downstream targets with observed changes in gene expression. Those with activation Z-scores ≥2 or ≤2 were considered “activated” or “inhibited”, respectively.

#### Isolation of metabolites from tumour tissue

Metabolites were isolated from IPL and SC tumours using a multiple-step homogenisation protocol. The excised tissue (≥8.8 ≤17.4 mg, wet weight) was transferred to a 2 mL polypropylene cryotube chilled on dry-ice and initially disrupted by three cycles of rigorous agitation at 6,500 rpm/20 s, using a Precellys Cryolys homogeniser (Bertin Technologies). 50:50 MeCN:water was added to the partially homogenised tissue and agitated repeatedly at 6,500 rpm/20 s rounds, until the sample was homogeneous. An additional 50:50 MeCN:water was added and the homogenisation repeated. Next, the suspension was frozen in liquid nitrogen and lyophilised to dryness. To produce a finer suspension for metabolite extraction, ceramic beads were added to the tubes containing the dried homogenate and milling was undertaken with repeated rounds at 6,500 rpm/20 s in the homogeniser.

Metabolites were extracted from the suspension as follows. Working on ice, two further rounds of milling were undertaken, firstly after the addition of 50 μL of LC–MS grade methanol, and again after a second 50 μL methanol addition. The 100 μL suspension was then topped up to 1,500 μL with cold methanol, mixed thoroughly by vortex and the cell debris was collected by centrifuge at 10,000 g for 15 min at 10°C. The supernatant was transferred to a fresh tube, from which a volume equivalent to 1 mg of homogenate was taken for gas chromatography/quadrupole time-of-flight spectrometry (GC-qTOF-MS) analysis (See below). For quality control purposes, a second 200 μL aliquot from each extract was combined into a single pooled sample, which was mixed by vortex and divided into replicate volumes (Pooled Quality Control, QC). All metabolite aliquots were dried in preparation for derivatisation, by vacuum concentration in an Eppendorf Concentrator Plus vacuum concentrator (Eppendorf, South Pacific Pty. Ltd., North Ryde, Australia).

#### Gas chromatography/quadrupole time-of-flight spectrometry (GC-qTOF-MS)

Metabolites were prepared for GC-qTOF-MS analysis by derivatisation to their methoxime and silyl derivatives as previously described by (Gummer et al., 2013), with modification. To the dried metabolites were added 100 μL of methoxylamine HCl (Sigma-Aldrich, Castle Hill, NSW, Australia) [20 mg.mL^−1^ in pyridine (UNIVAR)], followed by brief mixing by vortex and incubation at 30°C for 90 min with agitation at 900 rpm in an Eppendorf thermomixer. An aliquot of 20 μL was then taken and added to 40 μL of MSTFA (Sigma-Aldrich, Castle Hill, NSW, Australia) in a 200 μL glass analytical vial insert; 5 μL of n-alkanes [(C10, C12, C15, C19, C22, C28, C32 and C36); Sigma-Aldrich] in hexane] was added, mixed briefly by vortex, sealed by crimp-cap in a glass analytical vial, and incubated at 75°C for 30 min within a 7890 GC Oven (Agilent Technologies). Samples were cooled to room temperature, mixed briefly by vortex and loaded onto the GC-autosampler in a randomized sequence for analysis. The sequence was initiated with the injection of two preparative blank controls, followed by eight QC samples, with the sample set then interspaced with the analysis of a single PQC injection after every fourth sample, and finishing with four consecutive PQCs followed by four preparative blanks.

One microlitre of derivatised sample was injected into the inlet of a 7890 Gas Chromatograph (GC, Agilent Technologies, Santa Clara USA) operating at 230°C and a pressure of 11.138 psi in a splitless mode of operation. The GC was equipped with an Agilent VF-5ms column (Agilent Technologies, Santa Clara USA) with a temperature gradient beginning at 70°C, held for 1 min, before increasing at a rate of 5.63°C/min to a final temperature of 320°C, which was held for 10 min. Helium was used as the carrier gas, at a flow rate of 0.906 mL.min^−1^ and the retention time calibrated using standard n-alkanes. The sample syringe was washed with heptane after each injection. The GC was coupled to a 7200B quadrupole-time-of-flight (QTOF) mass spectrometer (Agilent Technologies, Santa Clara USA) installed with an EI ion source running at 70 eV, and data were acquired using an extended dynamic range acquisition, with spectra collected at a 10 GHz acquisition rate.

#### Metabolomics data processing

The acquired data were imported into AnalyzerPro v5.5.0.7304 (SpectralWorks, Runcorn UK) for ion deconvolution, metabolite identification and data visualisation. To assign a quantifier ion to each deconvoluted analyte, individual ion peak areas were aligned into a data matrix using Ruby (Gummer et al., 2013), whereby only ions within the assigned quality control thresholds were accepted; consisting of a percent relative standard deviation (%RSD) of the peak area of the QCs ≤45, a mean peak area of QC ≥ 1×10^5^, and maximum peak area across all samples being ≤2×10^6^ (previously determined to be within the linear dynamic range for quantitation) were accepted into the data matrix. Subsequently any ions measured in all six preparative blanks, thus representing analytical artefacts of non-biological origin, or suffering from sample carry-over, were removed from the final matrix. A quantifier ion was selected for each deconvoluted analyte if it met each of the above criteria and had the lowest %RSD of any ion within the deconvolution event. All other ions were used as qualifier ions. Metabolites were identified using an in-house library of mass spectra with accompanied chromatographic retention times, spectral-matched within AnalyzerPro, and using the National Institute of Standards and Technology (NIST) database. Multivariate data analysis was performed using MetaboAnalyst v4.0 (Pang et al., 2020). Data were Log_10_-transformed and range-scaled prior to interrogation by PCA and PLS-DA. Random Forest were performed using the R package Boruta (Kursa and Rudnicki, 2010) (Degenhardt et al., 2019) and MetaboAnalyst. The most influential identified metabolites were further interrogated by Receiver Operating Characteristic Curve analysis, following Log_10_-transformation and mean-centre scaling, using MetaboAnalyst 4.0.

#### Binding affinity assays

The LanthaScreen™ TR-FRET binding assay was performed following the manufacturer's guidelines (PV4894, Invitrogen) as follows, 20 mM stock solution of the ligand or control in DMSO were serially diluted using 100% DMSO to produce a range of concentrations from 100 μM to 1 pM. The test compound solutions were mixed with the reaction buffer in a 1 (ligand) in 10 (reaction buffer) dilution for a final 2X ligand concentration. This was mixed with the reaction components (glutathione S-transferase (GST)-PPPARγLBD (0.5 nM)/, terbium (Tb)-labelled anti-GST antibody (5 nM) and Fluormone pan-PPAR green (5 nM for PPARγ; 20 nM for PPARα) at room temperature in a ratio of 8 μLl 2X ligand solution: 4 μLl of 4XFluormone: and 4 μLl 4X GST-PPPARγLBD/terbium (Tb)-labelled anti-GST antibody to produce a final reaction mixture volume of 16 μL. The concentration of DMSO in the final reaction mixture was 5%. The reaction mixture was kept at room temperature for 2 h prior to recording the TR-FRET emission spectra using a CLARIOstar Plus microplate reader (BMG Labtech). Terbium was excited at 340 nM. Results are expressed as the ratio of fluorescence intensity at 520 nm (Fluormone emission) and 490 nm (Tb emission). The assay was carried out using a 396 well plate with 12 μL of reaction mixture placed in the wells. For each compound three serial dilutions were prepared and measured separately. The IC_50_ and SEM values were determined using a sigmoidal dose-response equation with varying slope using GraphPad Prism.

#### Transcriptional reporter assays

A transfected transporter cell line HG5LN was used (Balaguer et al., 2001). These cells were cultured in Dubecco's modified Eagle's medium (DMEM-F12 Thermo Fisher Scientific) with phenol red and supplemented with 5% foetal calf serum, incubated at 37°C in a 5% CO_2_/95% air-humidified atmosphere. Luciferase assays were performed in DMEM-F12 (Thermo Fisher Scientific) without phenol red and supplemented with 5% dextran-coated charcoal-stripped FCS (test culture medium), puromycin (Thermo Fisher Scientific) 0.5 μg/mL and geneticin (Thermo Fisher Scientific) 1 mg/mL. Cells were seeded at a density of 5 x 10^4^ cells/well and incubated for 8 h at 37°C in a 96-well plate white opaque tissue culture plates (Becton-Dickinson). Tested compounds were added in serial dilutions and incubated for 16 h at 37°C. After incubation, the medium containing the effectors was carefully removed and replaced by culture medium containing 3 x 10^−4^ mol/L luciferin (Sigma Chemicals). After 5 min incubation at room temperature, the 96-well plate was then read with a Microbeta Wallac luminometer and the luminescence was measured in the living cells for 2 s. All experiments were performed in quadruplicate.

#### Soft agar colony formation

Cells were incubated with different concentrations of GW6471 (from 0.5 μM to 8 μM) for a soft agar colony formation assay (Horibata et al., 2015). Briefly, 3% 2-hydroxyethyl agarose (agarose, A4018; Sigma-Aldrich, Australia) was prepared as stock solution. The bottom layer was prepared by incubating 20% agarose gel in R10 complete medium, RPMI 1640 (Invitrogen) supplemented with 10% foetal calf serum (FCS; Life Technologies), 20 mM HEPES (Sigma-Aldrich), 0.05 mM 2-mercaptoethanol (pH 7.2; Merck, Kilsyth, Australia), 60 μg/mL penicillin (Life Technologies), 50 μg/mL gentamicin (David Bull Labs), for a final concentration of 0.6%, 2mL per well, at 4°C for 1 h to allow the mixture to solidify, then incubating at 37°C for at least 30 min before seeding the cells. The cell-containing layer (10% agarose gel in R10 complete medium for a final concentration of 0.3%, 1 mL per well) was prepared with a concentration of 1,000 cells/mL. 1 mL of cells was then transfer to each well. The feeder layer (10% agarose gel in R10 complete medium for a final concentration of 0.3%, 1 mL per well was prepared with different concentrations of GW6471; each different concentration was then added to each well on top of the cells. The 6-well plate was then incubated at 4°C for 15 min before incubating at 37°C with 5% CO_2_ for a week. A new feeder layer was added once per week until day 22.

Colony counting was performed by adding 1 mL of 0.005% crystal violet (C0775; Sigma-Aldrich, Australia) in PBS on top of each well and incubating at room temperature for 24 h. Pictures were taken and colonies counted with ImageJ (v1.52a).

#### Migration and matrigel invasion assay

Cells were harvested and seeded in Incucyte®ImageLock 96-well plates (Essen BioScience) at a density of 10 x 10^4^ cells/mL overnight. A scratch wound was performed on the confluent cells with the 96-pin IncuCyte WoundMaker Tool (Essen BioScience). For migration assays, cells were washed with PBS once before adding 100 μL media with GW6471 at indicated concentrations. For matrigel invasion assays, cells were washed with PBS once before adding 50 μL of matrigel basement membrane matrix (FAL356231; Corning, NY, USA) at 8 mg/mL; lastly 100 μL media with GW6471 at indicated concentrations were added on top of the matrigel. Wounded cells were incubated in the IncuCyte ZOOM® at 37°C with 5% CO_2_. Data were analysed using the Incucyte® Scratch Wound Cell Migration Software Module and calculating the Relative Wound Density (%) (RWD) for both migration and invasion assays.

#### MTT assay

Cells were harvested and seeded in 96-flat well plates (Corning) at a density of 5 x 10^4^ cells/mL overnight. Media was removed and 100 μL media with GW6471 or GW9662 at indicated concentrations was added to each well. Cell viability and toxicity were measured at 48 h. Cells were incubated with 50 μL of a 2 mg/mL solution of (3-(4,5-dimethyl-thiazole-2-yl)-2,5-biphenyl tetrazolium (MTT, Sigma-Aldrich) in PBS for 4 h and then exposed to 100 μL dimethyl sulfoxide (DMSO; Sigma-Aldrich). Cell viability was measured by absorption at 570 nm in a microplate spectrophotometer (Spectromax 250 plate reader). Results are shown as relative cell viability.

#### *In vitro* metabolic stability

The test compound (1 μM) was incubated at 37°C with mouse liver microsomes (XenoTech) at a protein concentration of 0.4 mg/mL. The metabolic reaction was initiated by the addition of a NADPH-regenerating system and quenched at various time points over a 60 min incubation period by the addition of acetonitrile. Control samples (without NADPH) were included (and quenched at 2, 30, 60 min) to monitor for potential degradation in the absence of cofactor. The degradation half-life and *in vitro* intrinsic clearance (*in vitro* CL_int_) in microsomes were calculated using the apparent first order degradation rate constant for substrate depletion (Obach, 1999).

#### Liquid chromatography-mass spectrometry (LC-MS) bioanalysis

Compound quantification was performed using a Xevo G2 QTOF (*in vitro* metabolic stability) using positive electrospray ionisation under MS^E^ mode or a Waters Xevo TQD (mouse exposure) mass spectrometer using positive electrospray ionisation multiple-reaction monitoring mode. Compounds were eluted using an acetonitrile-water/0.05% formic acid gradient at a flow rate of 0.4 mL/min operating over a 4 min cycle on a Waters Acquity UPLC using a Supelco Ascentis Express RP C8 column (50 × 2.1 mm, 2.7 μm). Extraction was performed by precipitation using acetonitrile (1:1 volume ratio for microsomes and 2:1 volume ratio for plasma samples) followed by centrifugation. Compound concentrations were quantified against calibration standards prepared in an appropriate matrix to match the test samples (e.g. plasma or microsomes).

#### GW6471 formulation preparation

ABT Formulation: On the evening prior to dosing, ABT was dissolved in a 0.9% (w/v) saline using vortexing and sonicating, resulting in a colourless solution. The nominal concentration of ABT in the formulation was 10 mg/mL. GW6471 Formulation: The final drug contains 40% final volume of PEG400 (Kollisolv PEG E 400, Sigma Aldrich), and 60% final volume of Solutol HS-15 (Kolliphor HS-15, Sigma Aldrich). Solutol is previously mixed in a saline solution (12.5% concentration). On the day of dosing, the solid GW6471 was first dissolved in PEG400by thoroughly vortexing and sonicating for 5 min. Then, the saline solution containing Solutol HS-15 12.5% was added. Again, the formulation was then thoroughly vortexed and sonicated to produce a hazy pale yellow suspension with an apparent pH of 3.4. The nominal concentration of GW6471 in the formulation was 2 mg/mL. The bulk formulation was mixed by inverting the tubes prior to drawing each dosing volume. Animals were dosed within 1 h of formulation preparation. The dose administered to each mouse was calculated on the basis of the mouse body weight (determined prior to dosing).

#### Binding of GW6471 in mouse plasma and media assay

Protein binding was determined via rapid equilibrium dialysis (RED) with incorporation of a pre-saturation step (Charman et al., 2020). The pre-incubation process involved RED system being exposed to fresh aqueous solutions of GW6471 (400 nM) for three periods (including one overnight). For the protein binding determination, GW6471 was spiked into the diluted mouse plasma (10% (v/v); in PBS) or DMEM/F-12 media containing 5% dextran-coated charcoal-stripped FCS (at 2000 nM) and dialysed against protein-free PBS (for plasma) or base media (DMEM/F-12) containing GW6471 at 40 nM for 24 h at 37°C under ambient atmosphere on a plate shaker. Concentrations of GW6471 in dialysate and donor samples collected at the end of the dialysis period were determined via LC–MS.

#### Data analysis for binding of GW6471

Compound binding was assessed on the basis of the measured concentrations in dialysate and donor samples at the end of the dialysis period.

Based on the post-dialysis measured concentrations in donor (C_total_post_dialysis_) and dialysate (C_unbound_) for each RED unit, the unbound fraction of compound in assay matrix (*f*_*u*_diluted_) was calculated according to Equation 1 below:

(Equation 1) $$f_{u}{=}_{\;\frac{C_{\text{unbound}}}{C_{\text{total}\_\text{post}-\text{dialysis}}}}$$

For media, the value for *f*_*u*_ calculated according to Equation 1 above was used without further correction. The measured *f*_*u*_ value in diluted mouse plasma was further corrected for the dilution factor to determine the unbound fraction in neat mouse plasma according to Equation 2 per standard methods (Kalvass and Maurer, 2002):

(Equation 2) $$f_{u\_\text{undiluted}\;=\;\frac{1/D}{\left(\left(\frac{1}{f_{u\_\text{diluted}}}\right)-1\right)+1/D}}$$

Where *D* is the dilution factor (i.e. *D* = 10 for 10% diluted assay matrix).

#### Immunohistochemistry

Tissues were fixed with 4% paraformaldehyde for 48–72 h and embedded in paraffin. 5 μm sections were cut by microtome (Thermo Fisher Scientific) and placed on Slide SuperFrost Plus White 1 mm (Thermo Fisher Scientific). Slides were deparaffinised as previously explained for hematoxylin and eosin staining. Antigen retrieval was performed with 10 mM citrate, pH6, at high pressure in a pressure cooker for 15 min and 5 min slow release. Sections were rinsed twice with TBST buffer (Tris-Buffer Saline with Tween, 20 mM Tris, 500 mM NaCl, and 0.05% Tween 20) for 5 min under agitation. Quench tissue peroxidase H_2_O_2_ was performed for 10 min at room temperature with 3% H_2_O_2_ in distilled H_2_O. The Vectastain® ABC kit, peroxidase (Rabbit IgG) (Vector Laboratories) was used for the following steps. Non-specific binding was performed with 1.5% Normal goat serum in PBS for 20 min at room temperature. Primary antibody Angptl4 (1:50) (409800, Invitrogen), was diluted in PBS/1.5% goat serum and incubated 1 h at room temperature. The secondary biotinylated anti-Rabbit IgG (1:200) (Vector Laboratories), was diluted in PBS and incubated for 30 min at room temperature. Vectastain ABC reagent (Vector Laboratories) was used as per manufacturer's instructions, mixing Reagent A with Reagent B (1:1) and incubating at room temperature 30 min prior use. To use, Vectastain ABC reagent was added for 30 min at room temperature. The DAB peroxidase substrate kit (Vector Laboratories) was prepared per manufacturer's instructions (2.5 mL distilled H_2_O + 1 drop buffer stock + 2 drops DAB stock + 1 drop hydrogen peroxide), this reagent was added for 3 min at room temperature before stopping the reaction with distilled H_2_O. Finally, counterstaining was performed for 30 s at room temperature with Mayer's Hematoxylin (Sigma-Aldrich) and the slides were dehydrated and mounted as previously explained for hematoxylin and eosin staining.

### Quantification and statistical analysis

GraphPad Prism software was used to determine statistical significance of differences between groups by Student's t-test (Mann-Whitney test) when comparing two groups. Two-way ANOVA with Dunnett's multiple comparisons test correction was used for multiple comparisons. A p value <0.05 was considered significant. Each figure legend contains all the statistical details on each experiment, including the specific statistical test for that assay, exact value of n, what n represents and dispersion and precision measures. RNA sequencing statistical details can be found in the section STAR Methods details under Bulk RNA-seq analysis.
