# Supplementary material for: PPARα and PPARγ activation is associated with pleural mesothelioma invasion but therapeutic inhibition is ineffective
Source: iScience. 2021 Dec 4;25(1):103571. doi: 10.1016/j.isci.2021.103571 (PMC8692993; doi:10.1016/j.isci.2021.103571)
Supplement: Document S1. Figures S1–S9 and Tables S1–S3 [file mmc1.pdf]

iScience, Volume *25*

Supplemental information

PPARα and PPARγ activation is associated with pleural mesothelioma invasion but therapeutic inhibition is ineffective

M. Lizeth Orozco Morales, Catherine A. Rinaldi, Emma de Jong, Sally M. Lansley, Joel P.A. Gummer, Bence Olasz, Shabarinath Nambiar, Danika E. Hope, Thomas H. Casey, Y. C. Gary Lee, Connull Leslie, Gareth Nealon, David M. Shackleford, Andrew K. Powell, Marina Grimaldi, Patrick Balaguer, Rachael M. Zemek, Anthony Bosco, Matthew J. Piggott, Alice Vrielink, Richard A. Lake, and W. Joost Lesterhuis

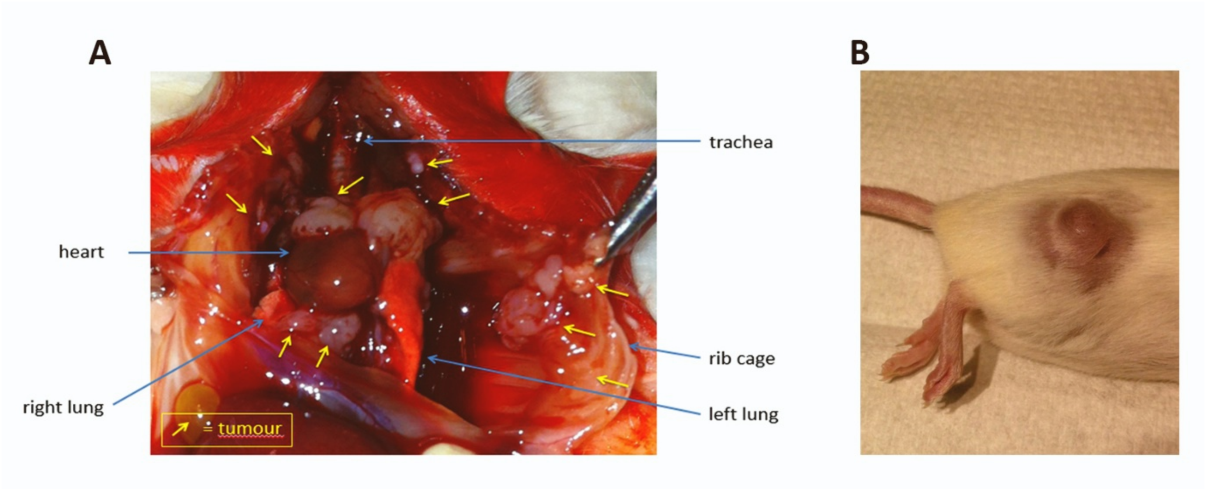

Figure S1 (Related to Figure 1). Tumours derived from mesothelioma cell lines are more invasive and proliferative in the pleural compared to the subcutaneous space. (A) Intraperitoneal AB1 tumour on day 10 in BALB/c mouse. Blue arrows point to different organs, yellow arrows point to tumour. (B) Subcutaneous AB1 tumour on day 10 in BALB/c mouse before being harvested.

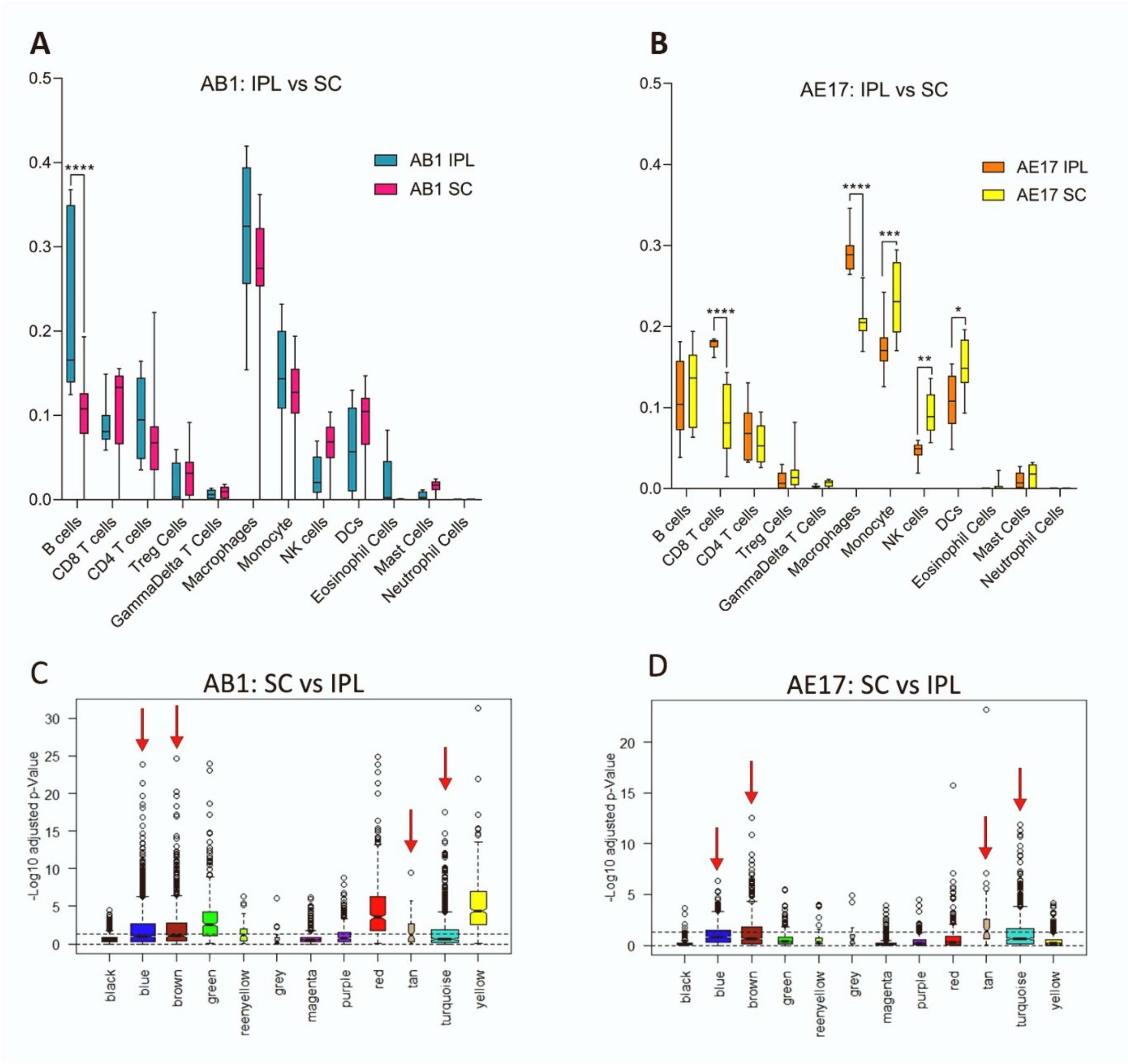

Figure S2 (Related to Figure 2). PPARα and PPARγ are identified as key regulators in invasive tumour development. (A and B) CIBERSORT analysis on AB1 and AE17 models. (A) AB1 model for IPL tumours vs SC tumours. B cells are significantly different; $p < 0.0001$. (B) AE17 model for IPL tumours vs SC tumours. $n = 8$. Two-way ANOVA with Sidak's multiple comparison (* $p < 0.02$, ** $p < 0.008$, *** $p = 0.0002$, **** $p < 0.0001$). (C and D) WGCNA analysis on (C) AB1 and (D) AE17 models comparing SC vs IPL tumours. Red arrows indicate the four significant models in both AB1 and AE17 models.

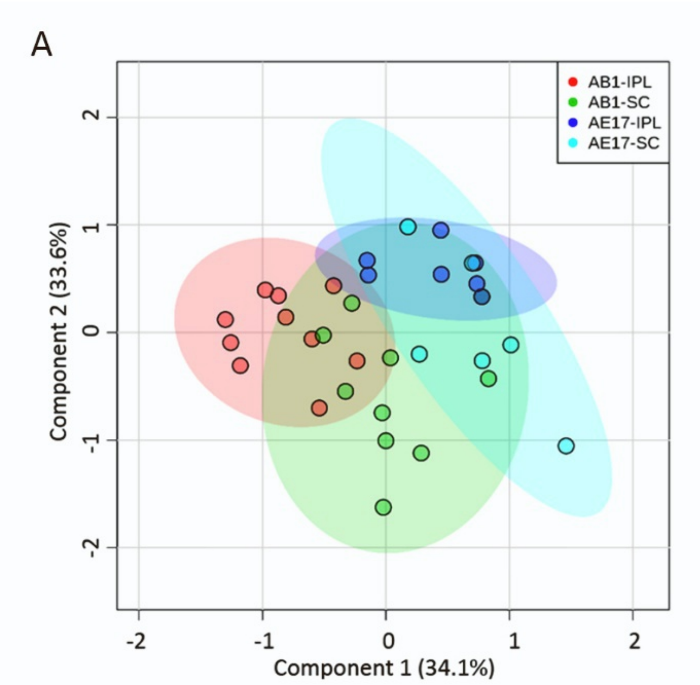

Figure S3 (Related to Figure 3). PCA identifies major source of variance within tumours. (A) Unsupervised modelling using Principal Component Analysis (PCA) identified the major source of variance within the metabolomes of intrapleural (IPL) and subcutaneous (SC) tumours being consistent with cell line, AB1 or AE17. Group differences due to tumour location are more subtly described within the displayed groupings.

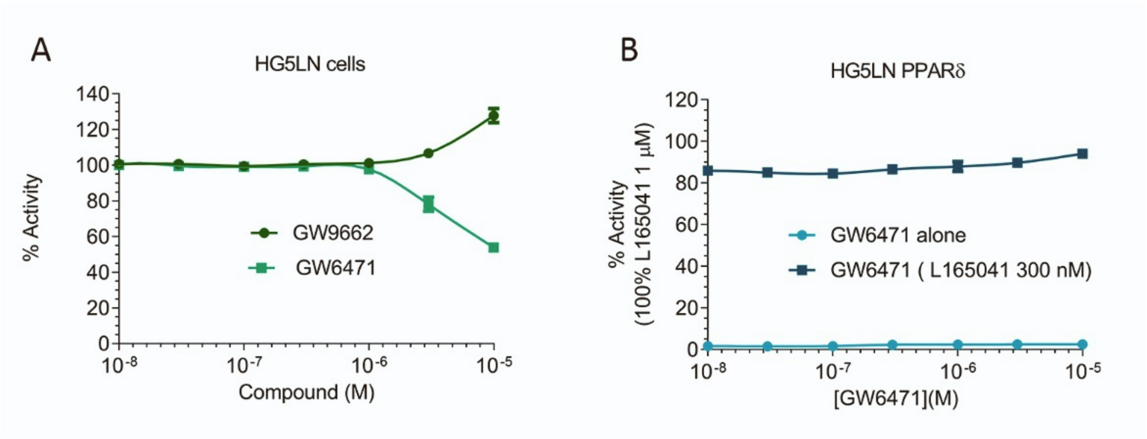

Figure S4 (Related to Figure 4). Use of parental HG5LN for GW6471 on PPARδ (A) Parental HG5LN cells enable to test the non-specific modulation of luciferase expression. (B) Reporter cell assay with HG5LN cells for GW6471 on PPARδ. The curve is presented as a non-linear regression; log[ligand] *vs* response. Data are presented as means ± SD values.

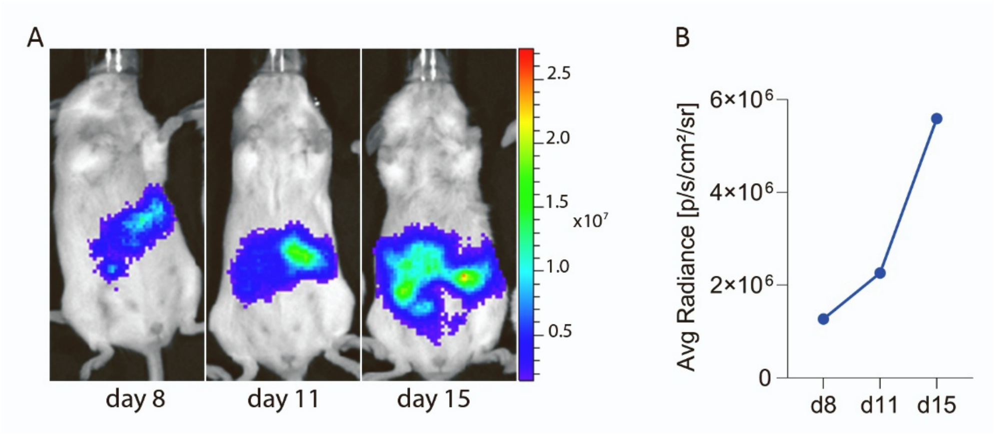

Figure S5 (Related to Figure 6). In vivo imaging system (IVIS) allows measuring AB1-Luc IP tumours over time. (A) AB1-Luc bearing BALB/c mouse is imaged via IVIS on day 8, 11 and 15. Coloured bar shows average radiance (p/s/cm^2/sr). (B) Average radiance is showed at 3 different time points representing tumour growth from day 8 to day 15.

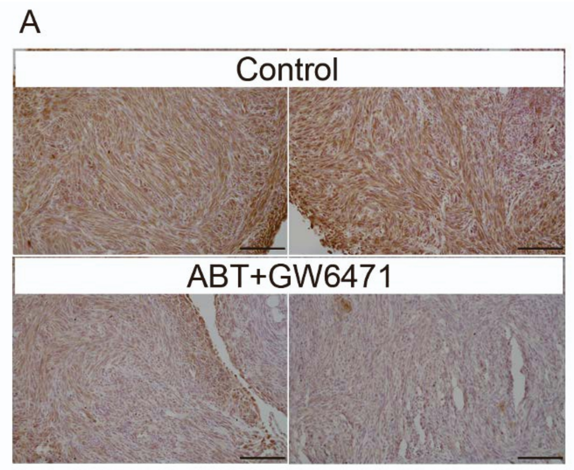

Figure S6 (Related to Figure 6). Angptl4 levels in AB1 mesothelioma tumours from mice treated with ABT and GW6471 or vehicle control. (A) Representative Angptl4 staining on control tumour samples and ABT+GW6471 treatment tumour samples. Scale bar = (x10) 100 µm. n = 14.

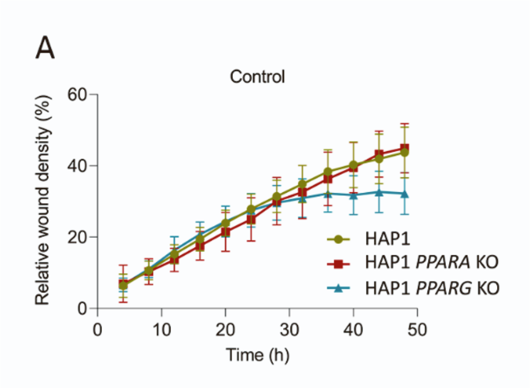

Figure S7 (Related to Figure 7). Genetic deletion of *PPARG* reduces migration capacity *in vitro.*

(A) Wound migration assay for HAP1, HAP1 *PPARA* KO, and HAP1 *PPARG* KO.

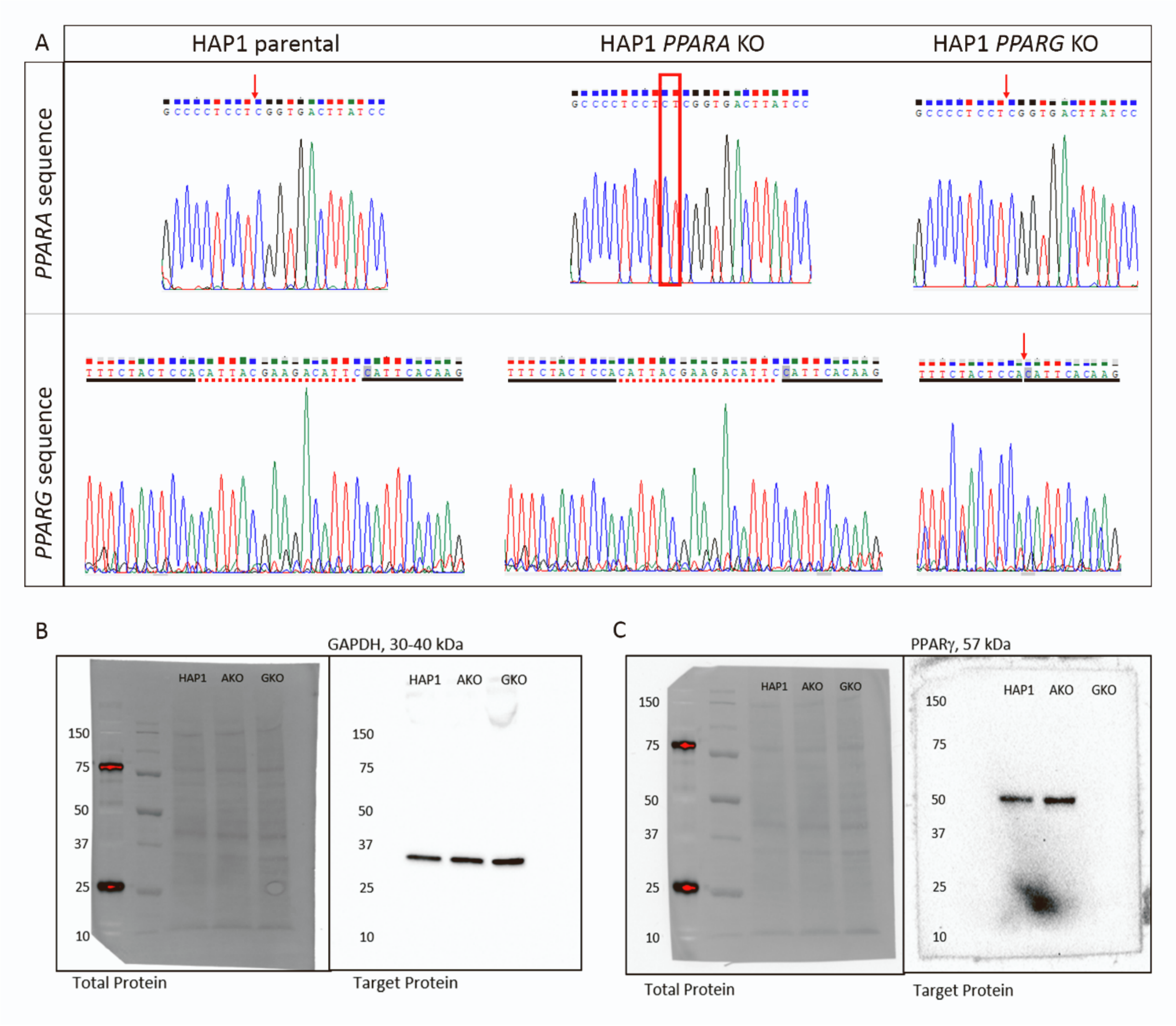

Figure S8 (Related to Figure 3, 4 and 7). Sanger sequencing and western blot confirms *PPARA* and *PPARG* knock out on HAP1 cell lines. (A) Chromatograms of HAP1 parental, HAP1 *PPARA* KO, and HAP1 *PPARG* KO. On the *PPARA* sequence, the red arrows show where the 2 bp insertions occurs for the HAP1 *PPARA* KO cell line. On the *PPARG* sequence, the red dotted line is the section that was deleted from the HAP1 *PPARG* KO cell line, also represented with a red arrow in the HAP1 *PPARG* KO cell line. (B) Total protein membrane and target protein GAPDH are shown for HAP1, HAP1 *PPARA* KO (AKO) and HAP1 *PPARG* KO (GKO) cell lines). (C) Total protein membrane and target protein PPARγ are shown for HAP1, AKO and GKO cell lines.

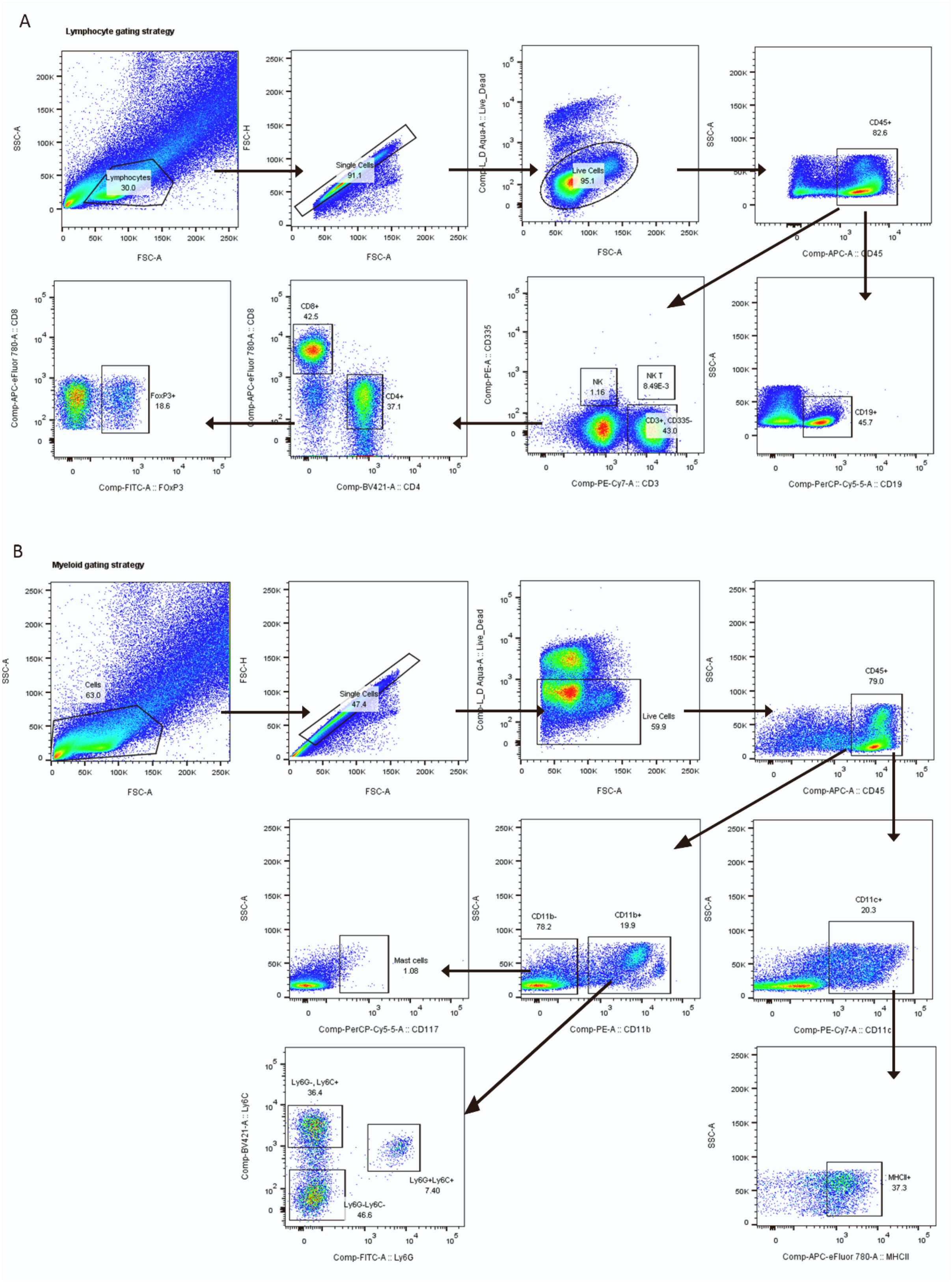

Figure S9 (Related to Figure 2). Gating strategy for lymphoid and myeloid panels for flow cytometry on SC and IPL tumours. (A and B) Gating strategy for (A) lymphoid and (B) myeloid panels for SC and IPL tumours at day 10.

Table S1 (Related to Figure 6). Protein binding data for GW6471 in mouse plasma and assay media (DMEM / F-12 media with dextran-coated charcoal-stripped 5% FCS).

Compound	Assay matrix	Fraction unbound (f_u)
GW6471	Mouse plasma	0.00059 ± 0.00005
	Assay media	0.0037 ± 0.0006

Note: Fraction unbound values are presented as the mean ± SD of n=3 independent dialysis units

Table S2 (Related to Figure 2 and S9). Flow cytometry markers and fluorochromes.

Marker	Fluorochrome	Distributor	Dilution	Clone	Catalogue No; RRID
**Antibodies for lymphoid panel**					
FVD	Zombie UV	BioLegend	1:2000		423107
CD45	APC	BioLegend	1:500	30-F11	103112; RRID:AB_312977
CD4	BV421	BioLegend	1:500	GK1.5	100438; RRID:AB_11203718
CD8a	APC-eFluor 780	eBioscience	1:200	53-6.7	47-0081-82; RRID:AB_1272185
FoxP3	FITC	eBioscience	1:100	FJK-16s	11-5773-82; RRID: AB_465243
CD335	PE	BioLegend	1:200	29A1.4	137604; RRID:AB_2235755
CD19	PerCP-Cy5.5	BioLegend	1:500	1D3/CD19	152405; RRID:AB_2629814
CD3	PE-Cy7	BioLegend	1:200	17A2	100219; RRID:AB_1732068
**Antibodies for myeloid panel**					
FVD	Zombie UV	BioLegend	1:2000		423107
MHCII	APC-eFluor 780	eBioscience	1:500	M5/114.15.2	47-5321-82; RRID:AB_1548783

CD11c	PE-Cy7	eBioscience	1:200	N418	25-0114-82; RRID:AB_469590
CD11b	FITC	BioLegend	1:500	M1/70	101205; RRID:AB_312788
Ly6G	PE	BioLegend	1:500	1A8	127607; RRID:AB_1186104
Ly6C	BV421	BioLegend	1:300	HK1.4	128032; RRID:AB_2562178
CD117	PerCP-Cy5.5	BD Biosciences	1:300	2B8	560557; RRID:AB_1645258
CD45	APC	BioLegend	1:500	30-F11	103112; RRID:AB_312977

Table S3 (Related to STAR Methods). Software or algorithms used in this paper

SOFTWARE OR ALGORITHM	SOURCE	IDENTIFIER
PKSolver Version 2.0	Zhang et al., 2010	doi:10.1016/j.cmpb.2010.01.007
Image Lab Software	Bio-Rad Laboratories	https://www.bio-rad.com/en-au/product/image-lab-software?ID=KRE6P5E8Z
Flowjo	Becton, Dickinson and Company	https://www.flowjo.com
FastQC v0.11.3	Andrews, n.d.	https://www.bioinformatics.babraham.ac.uk/projects/fastqc/
HISAT2 v2.0.4	Kim et al., 2015	doi: 10.1038/nmeth.3317
SummerizeOverlaps	Lawrence et al., 2013	doi: 10.1371/journal.pcbi.1003118
Stringtie v1.3.0	Pertea et al., 2015	doi: 10.1038/nbt.3122
Ballgown	Frazee et al., 2015	doi: 10.1038/nbt.3172
SAMStat v1.5.2	Lassmann et al., 2011	doi: 10.1093/bioinformatics/btq614
CIBERSORT	Newman et al., 2015	doi: 10.1038/nmeth.3337
DESeq2	Love et al., 2014	doi: 10.1186/s13059-014-0550-8
WGCNA	Langfelder and Horvath, 2008	doi: 10.1186/1471-2105-9-559
DCGL	Yang et al., 2013	doi: 10.1371/journal.pone.0079729
Ingenuity Systems	Krämer et al., 2014	doi: 10.1093/bioinformatics/btt703
AnalyzerPro v5.5.0.7304	SpectralWorks	https://spectralworks.com/software/analyzerpro/
MetaboAnalyst v4.0	Pang et al., 2020	doi: 10.3390/metabo10050186
Boruta	Kursa and Rudnicki, 2010, Degenhardt et al., 2019	doi: 10.18637/jss.v036.i11<br>doi: 10.1093/bib/bbx124

GraphPad Prism	GraphPad Software	https://www.graphpad.com/scientific-software/prism/
ImageJ v1.52a	Schneider et al., 2012	https://imagej.nih.gov/ij/<br>doi:10.1038/nmeth.2089
IncuCyte ZOOM v6.2.9200.0	Essen BioScience Inc.	https://www.essenbioscience.com/en/resources/incucyte-zoom-resources-support/software-modules-incucyte-zoom/
